# Effects of sea-level rise on physiological ecology of populations of a ground-dwelling ant

**DOI:** 10.1371/journal.pone.0223304

**Published:** 2020-04-17

**Authors:** L. M. Hooper-Bùi, R. M. Strecker-Lau, D. M. Stewart, M. J. Landry, A. M. Papillion, S. N. Peterson, R. A. Daniel

**Affiliations:** 1 Department of Environmental Sciences, College of Coast and Environment, LSU, Baton Rouge, LA, United States of America; 2 Feinberg School of Medicine, Northwestern University, Chicago, IL, United States of America; 3 Department of Nutritional Sciences, University of Texas, Austin, TX, United States of America; 4 Division of Clinical Immunology and Rheumatology, University of Alabama Birmingham, Al, United States of America; Universidade de Lisboa, Faculdade de Ciências, PORTUGAL

## Abstract

**Introduction:**

Sea-level rise is a consequence of climate change that can impact the ecological and physiological changes of coastal, ground-dwelling species. Sea-level rise has a potential to inundate birds, rodents, spiders, and insects that live on the ground in coastal areas. Yet, there is still much to be learned concerning the specifics of these impacts. The red imported fire ant *Solenopsis invicta* (Buren) excavates soil for its home and is capable of surviving flooding. Because of their ground-dwelling life history and rapid reproduction, fire ants make an ideal model for discovery and prediction of changes that may be due to sea-level rise. There are up to 500,000 individuals in a colony, and these invasive ants naturally have a painful sting. However, observations suggest that colonies of fire ants that dwell in tidally-influenced areas are more aggressive with more frequent stings and more venom injected per sting (behavioral and physiological changes) than those located inland. This may be an adaption to sea-level rise. Therefore, the objective of this study is to elucidate differences in inland and coastal defensiveness via micro-dissection and comparison of head width, head length, stinger length, and venom sac volume. But first because fire ants’ ability to raft on brackish tidal water is unknown, it had to be determined if fire ants could indeed raft in brackish water and examine the behavior differences between those flooded with freshwater vs. saltwater.

**Methods:**

To test the coastal-aggression hypothesis, inland colonies and coastal colonies, which experience relatively greater amounts of flooding, specifically regular tidal and windblown water and oscillations (i.e. El Nińo Southern Oscillation) from the Gulf of Mexico, were collected. To mimic sea-level rise, the colonies were flooded in salinities that correspond to both their collection site and conditions found in a variety of locales and situations (such as storm surge from a tropical storm). Individual ants were immediately taken from each colony for dissection before flooding, 1-hour into flooding, and 24-hours into flooding.

**Results and discussion:**

Fire ants use their venom to defend themselves and to communicate alarm or aggression. Dissections and measurement of heads, venom sacs, and stingers revealed both coastal and inland colonies experience an increase in venom sac volume after 24 hours; in fact coastal colonies increased their venom volume by 75% after 24 h of flooding Whether this venom sac enlargement is due to diffusion of water or venom sac production is unknown. These ground-dwelling ants exhibit physiological and behavioral adaptations to ongoing sea-level rise possibly indicating that they are responding to increased flooding. Fire ants will raft on high-salinity water; and sea-level rise may cause stings by flooded ants to be more severe because of increased venom volume.

## Introduction

Global climate change and subsequent sea level rise have the potential to alter the ecology and physiology of land-dwelling animals. Global climate models predict sea level rise of 0.2 to 2.0 m by 2100, with the southeastern U.S. experiencing an accelerated rate of 1.2 cm per year of relative sea level rise [1]. Modifications at the species level in response to climate change can consist of changes in phenology and shifts in each species’ range and collective community [2–3]. Complex dynamics of species result in unpredictable shifts in population and community interactions [2]; however, Parmesan and Yohe (2003) have shown that some changes are predictable [4]. Behavioral and physiological alterations have been measured at increased temperatures in the predicted range of near-future climate change. Estay et al. (2009) found when they modelled existing data on stored products insect pests that an increased temperature of 2–3˚C can have a substantial shift in invertebrate migration and development [5]. Consequently, the increased temperature is well studied in insects, in particular, ants [6–8], but less attention has been paid to sea-level rise’s effect on coastal animals. It is known from Field et al. (2017) who predicted extinctions of seaside sparrow population as tides increase [9].

In this study, because red imported fire ants are well studied, *Solenopsis invicta* (Buren) were used as a model for impacts of sea-level rise on ground-dwelling animals. The ants’ ability to survive freshwater flooding [10] makes it ideal for studying the impacts of climate change via sea-level rise. This fire ant is native to South America [11–12]. The lack of natural predators and competing ant species, combined with *S*. *invicta*’s high reproduction rate and ability to survive freshwater flooding have allowed the red imported fire ant initially to invade the southeastern United States in large numbers and displace native fire ant species such as *Solenopsis xyloni* and *Solenopsis geminata* [11–13]. Since the ants’ invasion into Mobile, Alabama in the 1920s and 1930s, *S*. *invicta* has established itself as a dominant ant species in the southeast and expanded in the U.S. as far as California, Washington D.C., and Puerto Rico [11–15]. In the last 20 years, fire ants have spread to Australia and Asia [15].

The fire ant has an aggressive nature. Typically, when a fire ant colony’s mound is disturbed, the queen avoids the point of disturbance, whereas brood-tending workers flee the brood chamber with the brood and other workers aggressively defend the colony from invaders [11, 16–17]. Defending workers exhibit a complex range of aggressive behaviors such as rushing towards an intruder, biting, and stinging; these behaviors are mostly mediated by pheromones and are context-dependent. Only female workers sting, and they do so by first grasping the enemy’s skin with their mandibles, then pushing the stinger through the skin and slowly releasing venom from storage in the venom sac [11, 12, 18]. Mated females and queens have a stinger, but do not sting. Instead, the stinging apparatus serves a different function for queens, allowing the controlled and deliberate dispersal of recognition pheromones [19] and egg laying.

Unlike honey bees which can sting only once, the red imported fire ant is likely to cause multiple stings by either maintaining a grip on the skin with its mandibles and rotating the rest of the body to produce a circle of pustules or by stinging as it crawls along the enemy’s skin [11, 13]. Deslippe and Guo (2000) found that venom chemical composition differs in caste members and corresponds to age, size, and function in workers [18]. Further, in polymorphic ants, worker adult ants attain their size based upon larval nutrition. Large workers produce more alkaloids with a higher saturated: unsaturated ratio than small workers and intermediate-aged workers produce more venom than young or old workers [18].

When flooded, all sizes of fire ant workers are known to change their behavior and become more aggressive, i.e. higher amount of venom injected per sting [20] and have more venom volume available for stings [21]. Hooper-Bùi observed that fire ants living in the coastal flood zone are more aggressive than those located inland even when they are not experiencing flooding. In the days following Hurricane Katrina, people who walked through floodwaters experienced a greater quantity of stings and greater intensity of wheals on the skin [21]. The proximate change may be the result of changes in the ants’ physiology; venom volume was examined here and chemical composition needs to be investigated in the future. Therefore, the objective of this study was to elucidate differences in inland and coastal aggression via micro-dissection and comparison of head width, head length, stinger length, and venom sac volume (because fire ants are polymorphic). It was hypothesized that, based on Haight’s 2006 work [20] and Papillion et al’s 2011 work [21] that coastal ants would exhibit higher venom volumes when flooded. In this work, this is referred to as the coastal-aggression hypothesis. Before analyzing these differences, the first experiment examined whether ant colonies could form rafts in brackish water as experienced by colonies on the coast and investigated the behavior differences between fire ants flooded with freshwater vs. saltwater. Then, the ultimate ecological changes can be predicted.

## Materials and methods

### Collection of colonies for rafting study (Experiment 1)

Fire ant colonies were collected from inland sites so they would have no history of experience with salt water. Coastal sites were selected for colonies that would periodically be inundated with salt water. Five colonies were collected from a pasture on LSU’s campus (N30.413726, W-91.193312, Baton Rouge, La) and were designated as controls (Control 1, Control 2, Control 3, Control 4, & Control 5) to receive freshwater flooding treatments. At the same site, five additional colonies were collected and designated as inland colonies receiving saltwater (Salt 1, Salt 2, Salt 3, Salt 4, Salt 5) flooding treatments. We stored colonies in 18.9-liter buckets, which had a layer of Teflon covering the top 10 cm as a step to prevent ant escapes [21]. All collections for this study were made under permits: LNHP-10-063, 2011—LNHP-11-046, LNHP-12-047, LNHP-14-039 from the Louisiana Department Wildlife and Fisheries' Natural Heritage Program, which approved the collection of ants used in this study. Insects are not vertebrate animals which are under the auspices of the LSU Institutional Animal Care and Use Committee.

### Flooding for rafting in fresh vs. saline (Experiment 1)

**Fig 1:**
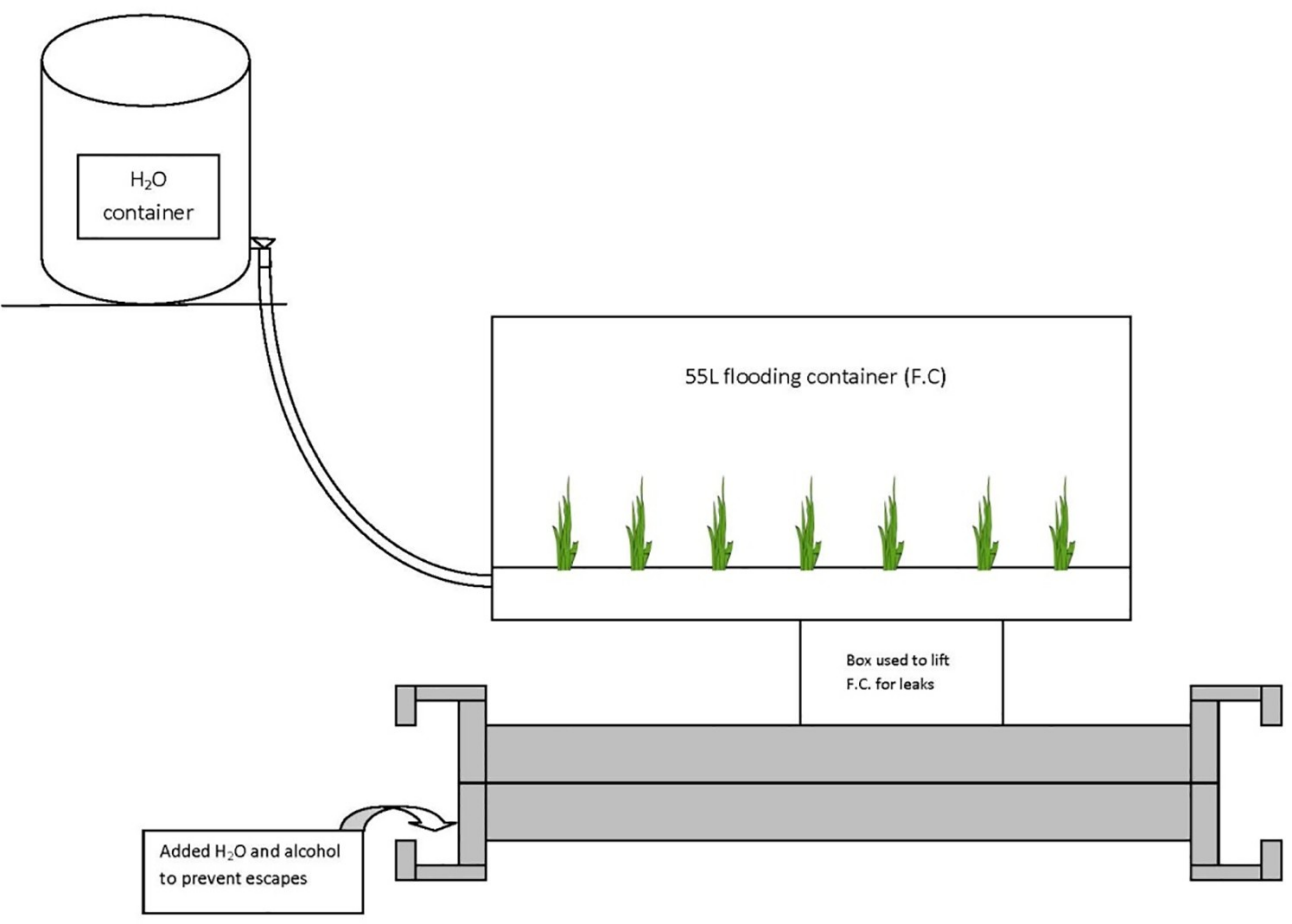
Fire ant flooding chamber where ants are flooded from below.

Colonies were transferred to a 55-L flood chamber the same day of collection. The colony was allowed to settle in the chamber for 24 h. The flood chamber (Fig 1) was self-constructed using an original design, which allowed colonies to be flooded from the bottom of the chamber simulating natural flooding conditions connected with sea-level rise. This result differs from Banks et al. (1981) and Chen (2007) who describe flooding from above [22].

The flooding chamber consisted of a 55-L Rubbermaid container, which had an irrigation tube affixed through the wall near the bottom with a weight holding it in place. The upper 10cm portion of the flood chamber had Teflon applied to the vertical sides to prevent escapes. Three, 3-mm holes were punctured into the portion of the tube that lay in the bottom of the chamber (Fig 1). Tubing extended out the side of the chamber (entrance of chamber was sealed with silicon) in order to attach the flood chamber to an elevated 30-L water tank (Nalgene). The tank was filled three-fourths to the top with deionized water for freshwater experiments. For at least 3 h, water flowed from the elevated tank to slowly flood the soil in the chamber via gravity, from the bottom-up. Flooding continued until the colony was no longer on any soil surface and completely rafting (as described in Adams et al. 2011) [13]. Proper precautions were taken during collecting and flooding to prevent escapes and personal injury as described in Nester [23].

Once the colony was flooded, talc powder was rubbed on the Teflon and rim of the flood chamber to prevent the ants from escaping in case the Teflon was wet [24]. A stick was secured in the middle of the container, so rafting colonies had something to tether to in order to prevent escape. The above-described process were repeated for the saltwater trials using water with a salinity of 10 ppt (Instant Ocean Salt Mix, Spectrum Brands, Blacksburg, Va) in the water tank, following the same methods. The salinity of 10 ppt was similar to that which the colonies on the coast of Louisiana experience (LMHB field measurements). Rafting behaviors were observed until rafts were no longer visible (the ants drowned) or bacterial plaque formed on the water, which allowed the flooded colonies an additional means of support. Upon experiment termination, the remaining ants were collected. individual brood (eggs, larvae, and pupae), workers, and whole rafts were observed under microscope and documented raft structure variations.

### Collection of coastal (Experiment 2) and inland (Experiment 3) colonies for venom volume studies

Thirteen coastal colonies were collected from Holly Beach in Cameron Parish, Louisiana (N29.771012, W-93.459272). Colonies were selected for medium to large (number of workers) with brood, but there was no discrimination for monogyne, polygyne, (single or multiple queen colonies) or queenless colonies because sorting through the colonies would have been disruptive. Three colonies (Holly Beach 4, Holly Beach 5, and Holly Beach 8) were collected approximately 200 meters from the high tide line (32.2°C air temperature, 6.63 knots wind speed, 26.8 ppt, 27.0°C water temperature). Five colonies (Holly Beach 10, Holly Beach 11, Holly Beach 12, Holly Beach 14, and Holly Beach 15) were collected approximately 300 meters from the high tide line (24.28°C air temperature, 3.28 knots wind speed, 25.28 ppt, 18.2°C water temperature). Five colonies (Holly Beach 18, Holly Beach 19, Holly Beach 20, Holly Beach 26, and Holly Beach 27; the numbers are not consecutive because not every collected colony was used because they were determined to be too small–or didn’t contain brood–after collection) approximately 200 meters from the high tide line (18.26°C air temperature, 2.7 knots wind speed, 25.0 ppt, 13.1°C water temperature). These colonies experienced inundation during storm surge, extreme high tides, and/or El Niño Southern Oscillation (ENSO) events.

Four additional *S*. *invicta* coastal colonies (Venice 1, Venice 2, Venice 3 and Venice 4) were collected from Tide Water Road in Venice, Louisiana (N29.232155, W-89.389314, air temp = 7.72°C, mostly sunny with a wind speed of 6.08 knots and 63% humidity), an area that experiences regular saltwater flooding via tidal and windblown water. These events are exacerbated by ENSO. Coastal colony Venice 5 was collected later from the same location under similar conditions (23.88°C, partly cloudy, 6.3 knots mph wind speed, 63% humidity). Two inland colonies (Inland 1 and Inland 2) from the Louisiana State University campus in Baton Rouge, Louisiana (30°C, partly cloudy, 6.08 knots wind speed, 68% humidity). The colony designated Inland 3 was collected from the same location at a different date (26°C, partly cloudy, 6.08 knots wind speed, 94% humidity).

All colonies were placed into plastic cylindrical buckets lined with Teflon to prevent escape, and transported them to Louisiana State University. Colonies were fed what would resemble a typical diet of frozen crickets and 20% honey water and temporarily stored in the Hooper-Bùi rearing room which was maintained at 19–27°C and 27–31% humidity.

### Flooding of coastal colonies (Experiment 2)

Colonies were transferred from the collection buckets into large, plastic flooding chambers lined with Teflon and allowed at least 24 h to acclimate. Ants were floated out of the soil in water that matched the salinity of their respective habitat using Instant Ocean. The above-described flooding chamber was used to separate the ants from the soil. All colonies rafted; ant colonies were removed from the flooding chamber immediately upon rafting, placed in soil-free containers, and allowed to acclimate for 24 h. Immediately before the experimental flooding, several ants were randomly chosen from the colony and placed in a centrifuge tube and chilled at 4°C until dissection (pre-flood control cohort). One liter of water was quickly added to the container containing colonies to simulate the rush of floodwater. Then, water was dripped at a constant rate of 1500 mL per hour using a hanging IV bag until ant colonies were again rafting. The drip rate of 1500 mL was used to mimic rainfall typical of southern Louisiana (= ~0.1–2.5 cm/h) or rising seawater.

Colonies in the experimental group received the same treatment; however, the amount of water added varied from a range of pre-determined saline solutions using Instant Ocean. Typical salinities tested were 8 ppt (common on the east side of the Mississippi River where there are freshwater diversions–LMHB unpublished data and CRMS data), 12 ppt (typically found in a saltwater marsh west of the Mississippi River–LMHB unpublished data and CRMS data), 18 ppt (that which flooded the marsh during TS Debbie–LMHB unpublished data and CRMS data), and 24 ppt (to mimic storm surge from a hurricane–LMHB unpublished data and CRMS data).

One hour into flooding, without disrupting the raft, >10 individuals were randomly chosen, from the rafting colony and immediately refrigerated until dissection (1-hour cohort). Seven and 24 h into flooding, >10 individuals were chosen from the raft and immediately refrigerated until dissection (designated 7-h and 24-h cohorts).

### Dissection of all flooded ants

Fire ants are polymorphic. In order to standardize the venom sacs across the different sizes, common landmarks (discussed below) were measured before dissection. Within one hour of collection, ants were removed from cold storage. Dissection was quickly performed under a stereomicroscope under low light, in order to not disturb the quiescent state of the chilled ants or cause desiccation of the venom sac. A flat scalpel, an angled scalpel, and dissection pins were used to remove the head, stinger and venom sac using methods similar to those described by Papillion et al. (2011) [21]. Digital calipers were used to measure head width from eye to eye, head length, stinger length from base to point, venom sac width, and venom sac length as described in Papillion et al. (2011) [21]. The volume of the tear-shaped venom sac was calculated from width and length using the equation $VSV=(VSL+\frac{VSL}{2})\;3*\frac{4}{3}*3.14$ A total of 778 individuals were dissected and included in this study including 229 coastal ants from Venice, 390 coastal ants from Holly Beach, and 159 inland ants. Sample sizes could not be standardized across the experiments because numerous venom sacs broke during dissection or measuring. Further, some venom sacs shriveled and those measurements were not included.

### Data analysis

Head width is established as a direct indicator of ant size and weight; it is used here to provide information on venom sac size of coastal and workers of different sizes [25]. Colonies were analyzed individually for differences in head width, venom sac volume, stinger length, and head length across cohorts that were collected immediately before the flood event, 1-h, 7-h, and 24-h into the flood event (see supplemental information for individual results). The Shapiro-Wilk test was used to test the normality of the head width, stinger length, and venom sac of each cohort. Cohorts that did not meet parametric assumptions were analyzed using the Mann-Whitney U test whereas those datasets that passed the normality test were subjected to T-tests. Due to the potential impact of Type 1 Error inflation, Bonferroni corrections were applied to all t-tests by multiplying the p-values by the number of tests conducted (in most cases 3) and by determining α_critical_ = 0.0492 (α_critical_ = 1 –(1 –α_altered_)^k^, where k = the number of comparisons on the same dependent variable). In each case, pre-flood measurements were analyzed against 1-h cohort measurements, 7-h cohort measurements, or 24-h cohort measurements. Data from individual colonies at each location were later pooled to form a complete data set.

To look for general trends, the complete data set was analyzed in the same manner as the individual colonies (S1–S4 Figs. S1–S8 Tables)) using head width, venom sac volume, stinger length, and head length. The complete coastal data were then separated by head width size into three categories designated small workers (head width < 0.75 mm), medium workers (head width 0.75 mm ≤ 1 mm) and large workers (head width ≥ 1 mm). In each of these three categories, the pre-flood, 1-h, 7-h, and 24-h cohorts of each size group were analyzed as described previously, using the Shapiro-Wilk test for normality, unpaired t-tests, Mann-Whitney U tests, and Bonferonni corrrections, when appropriate. The complete inland data were similarly grouped and analyzed in the same fashion. Coastal and inland complete data and size-grouped data were also analyzed such that venom sac volume was normalized to head width by the equation *r = vol/hw*, where *vol* is the venom sac volume in cubic millimeters, *hw* is the head width in millimeters, and *r* is a relative value that describes venom sac volume per unit head length. This method is similar to the size-standardization used by Haight and Tschinkel [24–25]. Linear regressions were performed for complete data as well as size-grouped data for both coastal and inland workers.

## Results

### Rafting study (Experiment 1)

Extraction of ants from the soil by flooding from the bottom-up rather than pouring water from the top resulted in the ants gradually moving out of flooded soil as they do in nature. The workers moved the members of the colony, particularly the eggs and brood (larvae and pupae), higher as the water rose and the soil became more saturated; the portions of the colony closest to the floodwater were evacuated by moving the eggs, brood, and queen(s) vertically. This method allowed the ants to naturally form a raft including all the brood, without any manipulation of the soil as described in Banks 1981 [22].

### Rafting in freshwater (Experiment 1)

When the ants were flooded with both freshwater and saltwater, ants were observed to use all floating objects within their environment to support and anchor the raft. Rafting anchors included the stick the researchers placed in the middle of the flood chamber, floating grass, or dead ants. Additionally, in the freshwater control trials, the bacterial plaque growth grew thickly from day two onward and covered the entire water surface area in the flood chamber. This plaque aided the colony in survival during the flood. The ants in all trials moved the brood to the highest surface above the rising water until the raft became the only option for colony survival. Brood were the foundation on which the raft was formed in both freshwater and saltwater. In a few cases, rafts that made it to the edge of the flooding chamber used brood to form a ladder over the vertical portion of the chamber that had Teflon and talc powder as a means of escape. The moisture on the brood and ants’ tarsi allowed them to stick to and climb the Teflon which underscored the importance of having a central structure to secure the raft.

The freshwater controls were observed to have one main raft or two separate, but sustainable, rafts. The colonies moved to the highest surface in the chamber until rafting became their only option for survival; sometimes rafting in multiple groups. Worker ants on the edge of the rafts were observed hanging onto the raft with their back legs and splaying their first two sets of legs. This allowed them to join other rafts or find high ground in nature. The colonies maintained their rafts although they shrunk in size over time as workers and brood dropped out and drowned. Once flooded, the freshwater colonies rafted with no difficulty. In several control colonies, a bacterial plaque (described above) formed on the water surface. The plaque consisted of a combination of bacterial colonies and mold. Eventually, the plaque would grow to the point of being so thick the ants could walk on top without compressing the meniscus of the water.

### Rafting in saltwater (Experiment 1)

In saltwater trials, the colonies stayed together immediately after tipping the raft into the water, forming rafts similar to those in the freshwater control. On day two following saltwater flooding, the colonies rafted in multiple smaller, separate rafts rather than one (or two) main mass(es) that are typically seen in freshwater-flooded ants. On day one of flooding with saltwater, brood were used as the foundation of the rafts, which allowed the colony to raft in a similar manner to the control colonies. As time progressed, the brood and a small number of workers gradually sank in the saltwater. On day two, close inspection of the separate rafts showed that the ants used dead workers as the foundation of the raft, rather than the brood. Eventually, most of the brood dropped from the bottom of the raft to the bottom of the flood chamber. As the brood drowned and dropped, the remaining floating portion of the colony switched to dead ants as a float and foundation of their raft on the surface of the water. When the saltwater rafted-brood were examined using microscopy, they were shriveled.

### Coastal results (Experiment 2)

**Fig 2:**
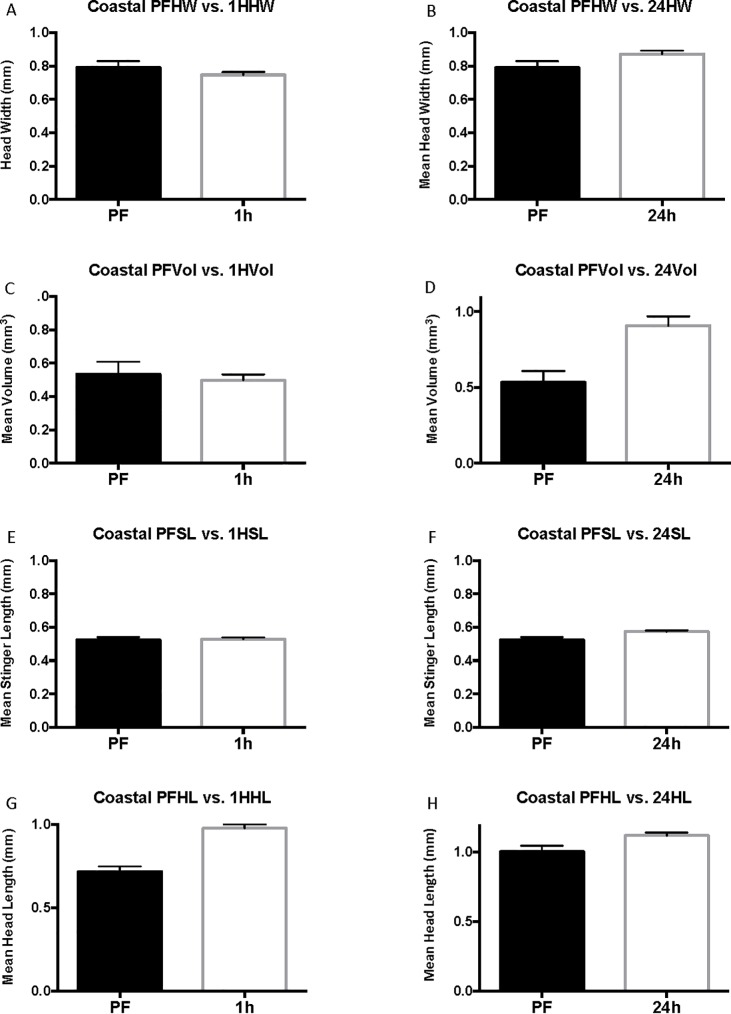
Complete coastal results. **Mean head width (HW), mean stinger length (SL), and mean head length (HL) are reported in mm.** Mean volume is in mm^3^. The error bars represent standard error of the mean. PF refers to pre-flood cohorts, 1h refers to 1-hour cohorts, and 24h refers to 24-hour cohorts.

Based on the previous experimental results that fire ant colonies can raft on saltwater for at least 24 hours, the effect of saltwater flooding on the volume of the venom sac was tested in coastal ant colonies. Individual colony results are presented in S1 Table and S1–S4 Figs. In flooded coastal colonies, the surviving worker body size was larger. In fact, individuals in the 24-hour coastal cohorts were larger than their pre-flood counterparts in all measured aspects, including venom sac volume (Fig 2). Head width was significantly greater by 10%, from 0.79 ± 0.038 mm (mean ± SEM) for pre-flood individuals to 0.87 ± 0.020 mm for 24-hour individuals (P = 0.0275, U = 1111) (Fig 2A and 2B). An increase in venom volume was seen in 24-hour cohorts, but not 1-hour and 7-hour cohorts (Fig 2C and 2D). Before flooding, the average (± SEM) venom sac volume was 0.53 ± 0.075 mm^3^, and after 24 hours mean venom sac volume was 0.91 ± 0.062 mm^3^ (P = 0.0002, U = 822) with an increase of 72%. Pre-flood workers had an average stinger length of 0.52 ± 0.017 mm, whereas 24-hour workers had significantly longer stingers with a mean of 0.57 ± 0.008 mm (9.6%, P = 0.0097, U = 1040) (Fig 2E and 2F). Head length was the only parameter for which a significant difference was seen for 1-hour and 24-hour cohorts. Head length was greater from 0.97 ± 0.023 mm pre-flood to 1.00 ± 0.044 mm after one hour (3%, P <0.0001, U = 976), then to 1.12 ± 0.020 mm after 24 hours (13%, P = 0.43, U = 990.5) (Fig 2G and 2H).

**Fig 3:**
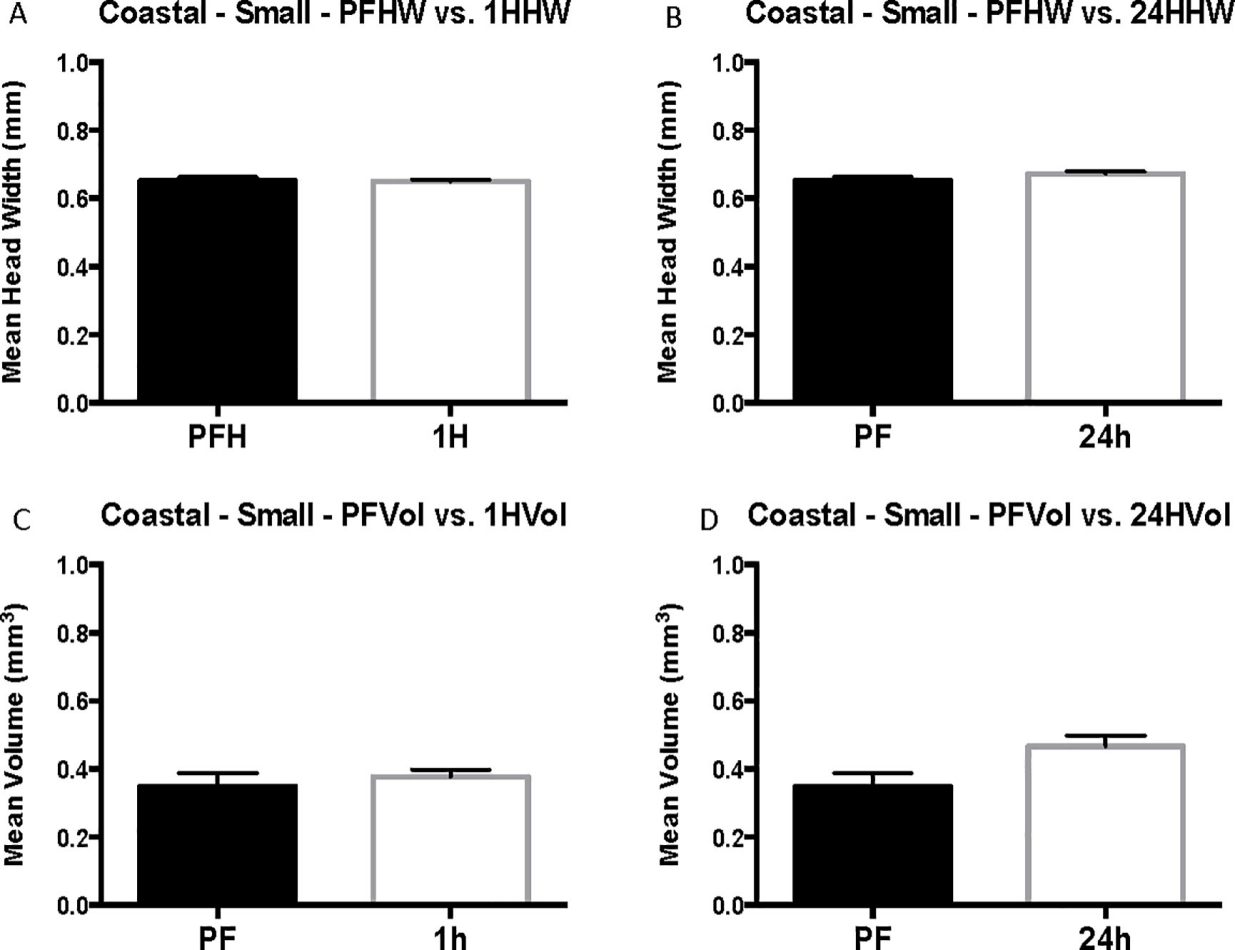
Small coastal head width and venom sac volume. Mean head width (HW) is in mm. Mean venom sac volume (Vol.) is in mm^3^. The error bars represent standard error of the mean. PF refers to the pre-flood cohort (n = 15), 1H refers to the 1-hour cohort (n = 46), and 24h refers to the 24-hour cohort (n = 39).

**Fig 4:**
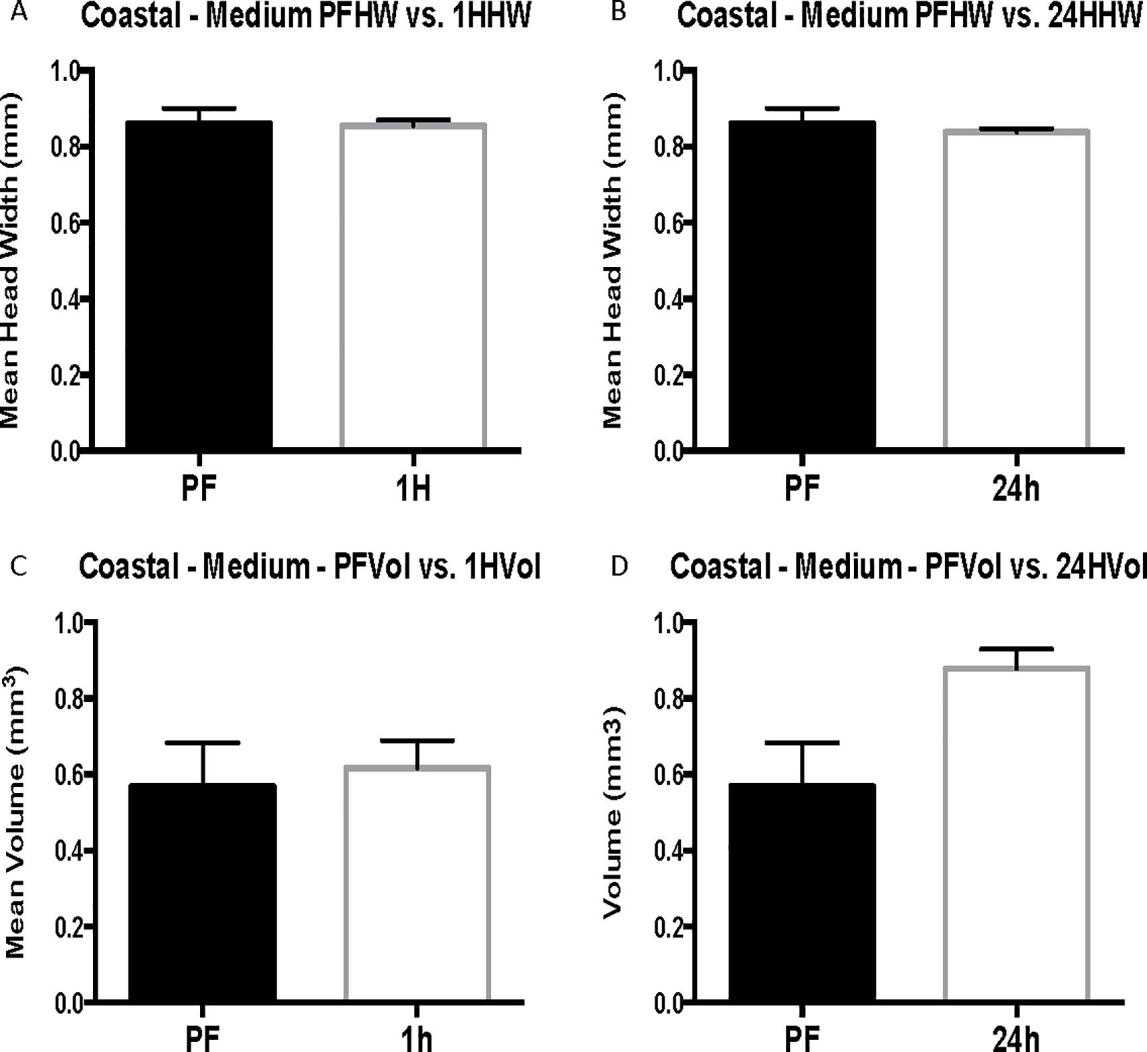
Medium coastal head width (HW) and venom sac volume (Vol). Mean head width is in mm. Mean venom sac volume is in mm^3^. The error bars represent standard error of the mean. PF refers to the pre-flood cohort (n = 6), 1H refers to the 1-hour cohort (n = 19), and 24h refers to the 24-hour cohort (n = 51).

**Fig 5:**
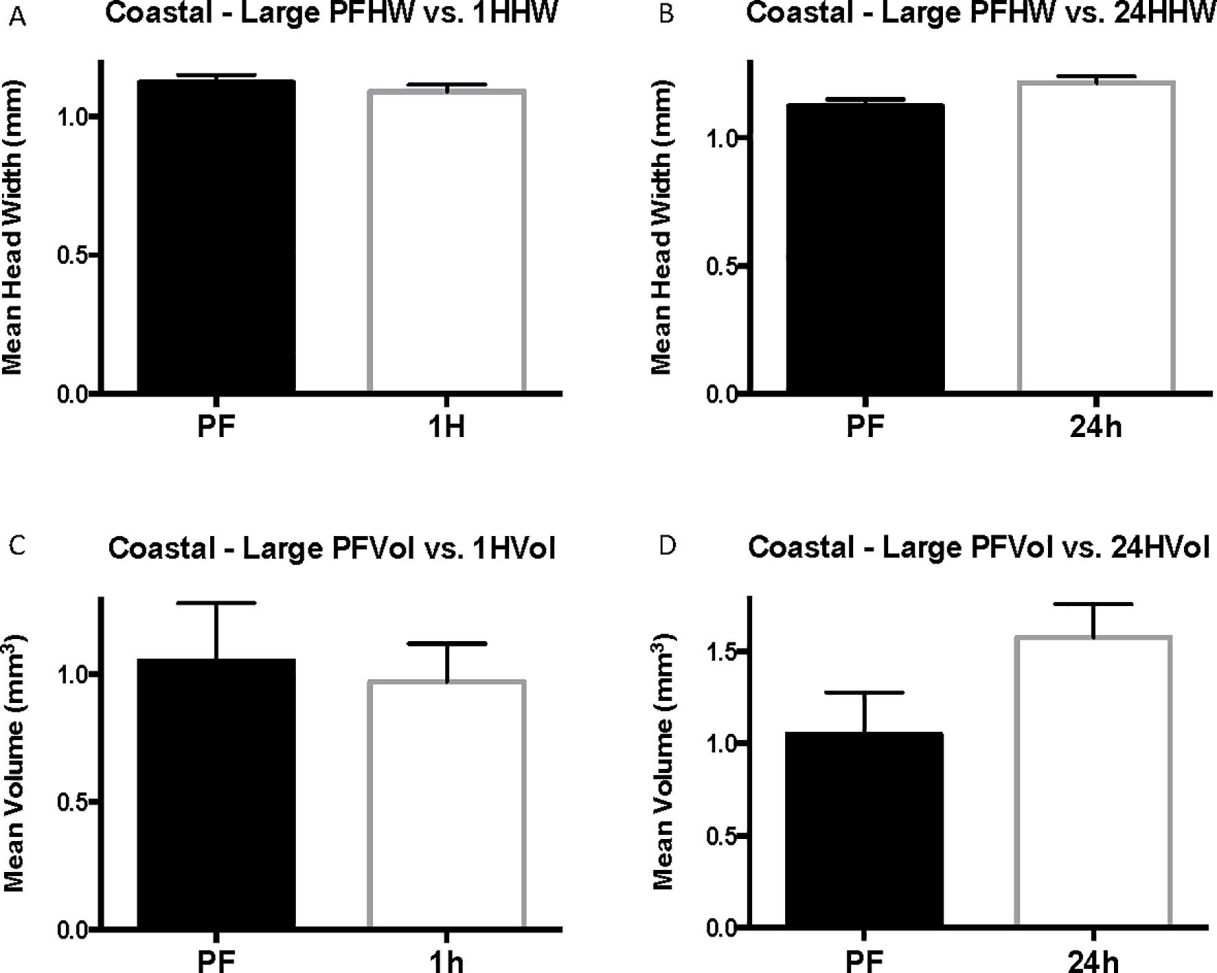
Large coastal head width (HW) and venom sac volume (Vol). Mean head width is in mm. Mean venom sac volume is in mm^3^. The error bars represent standard error of the mean. PF refers to the pre-flood cohort (n = 5), 1H refers to the 1-hour cohort (n = 7), and 24h refers to the 24-hour cohort (n = 28).

Grouping of coastal worker data by head-width size controls for differences in head width across cohorts and eliminates significant differences in head width for each size group. When coastal cohorts are grouped by head width, venom sac enlargement after flooding is observed in 24-hour cohorts of small and medium-sized workers but is not significant in large workers (head width > 1 mm) (Figs 3–5). The head width is essentially the same for pre-flood, 1-hour, and 24-hour cohorts of each size group (Table 1). Small workers (head width <0.75 mm, range = 0.57–0.71 mm, N = 100) had an average change in volume of 0.12 ± 0.056 mm^3^ with a 74% increase in venom sac volume from 0.35 ± 0.040 mm^3^ pre-flood to 0.47 ± 0.031 mm^3^ 24 hours into the flood (Corrected P = 0.120, t = 2.104, df = 52) (Fig 3). Medium workers (0.75 mm ≤ head width < 1 mm, range = 0.75–0.99, N = 76) also experienced significant venom sac enlargement. Medium workers had an average volume of 0.88 ± 0.051 mm^3^ after 24 hours compared with only 0.57 ± 0.115 mm^3^ for pre-flood medium workers for a 0.31 mm^3^ increase (P = 0.0714, U = 67.5) (Fig 4). This resulted in a 35% increase after 24 hours. Although the mean venom sac volume increased in large workers from 1.05 ± 0.228 mm^3^ in pre-flood cohorts to 1.58 ± 0.180 mm^3^ in 24-hour cohorts for a 33% increase, this increase is not significant because of the large variation in the dataset (corrected P = 0.6645, U = 45) (Fig 5).

**Table 1:** Fire ant head width and venom sac volumes (calculated) and results of statistical analysis when grouped by size. Bonferroni corrections were applied to the p-values used in multiple comparisons to reduce the possibility of a Type-1 error. Those p-values which were corrected are noted with a • symbol. Corrected p-values were compared to the Bonferroni-corrected α_critical_ = 0.049.

Factor(s) tested	Test	Mean ± SEM	Range	N	P	Different	t	U	df
Small Coastal Pre-flood head width		0.65 ± 0.010	0.57–0.71	15					
Small Coastal 1-hour head width	Unpaired t-test	0.65 ± 0.006	0.57–0.74	46	0.999•	No	0.1359		59
Small Coastal 24-hour head width	Mann-Whitney	0.67 ± 0.007	0.61–0.74	39	0.455•	No		218	
Small Coastal Pre-flood venom sac volume		0.35 ± 0.040	0.13–0.64	15					
Small Coastal 1-hour venom sac volume	Mann-Whitney	0.38 ± 0.021	0.19–0.84	46	0.999•	No		313	
Small Coastal 24-hour venom sac volume	Unpaired t-test	0.47 ± 0.031	0.13–1.05	39	0.120•	No	2.104		52
PF to 24h increase = 74%									
Medium Coastal Pre-flood head width		0.86 ± 0.040	0.76–0.99	6					
Medium Coastal 1-hour head width	Mann-Whitney	0.85 ± 0.016	0.75–0.97	19	0.999•	No		56	
Medium Coastal 24-hour head width	Mann-Whitney	0.84 ± 0.009	0.75–0.99	51	0.999•	No		1415	
Medium Coastal Pre-flood venom sac volume		0.57 ± 0.115	0.38–1.07	6					
Medium Coastal 1-hour venom sac volume	Mann-Whitney	0.62 ± 0.072	0.20–1.56	19	0.999•	No		50	
Medium Coastal 24-hour venom sac volume	Mann-Whitney	0.88 ± 0.051	0.22–2.06	51	0.071•	No		67.5	
PF to 24h increase = 70%									
Large Coastal Pre-flood head width		1.12 ± 0.027	1.06–1.21	5					
Large Coastal 1-hour head width	Mann-Whitney	1.09 ± 0.024	1.02–1.22	7	0.999•	No		12	
Large Coastal 24-hour head width	Mann-Whitney	1.21 ± 0.027	1.0–1.48	28	0.664•	No		44.5	
Large Coastal Pre-flood venom sac volume		1.05 ± 0.228	0.46–1.84	5					
Large Coastal 1-hour venom sac volume	Mann-Whitney	0.97 ± 0.148	0.31–1.53	7	0.999•	No		16	
Large Coastal 24-hour venom sac volume	Mann-Whitney	1.58 ± 0.180	0.51–4.78	28	0.664•	No		45	

PF to 24h increase = 66%

Size-grouped coastal unpaired t-tests and Mann-Whitney tests. Head width, stinger length, and head length are reported in mm. Volume is in mm^3^. N represents the number of workers within the corresponding group, P is the p-value, and U is U-value from Mann-Whitney U tests. Tests that determined significant (correct p < 0.049•) differences are marked by the word “yes” under the column labeled “different.”

**Fig 6:**
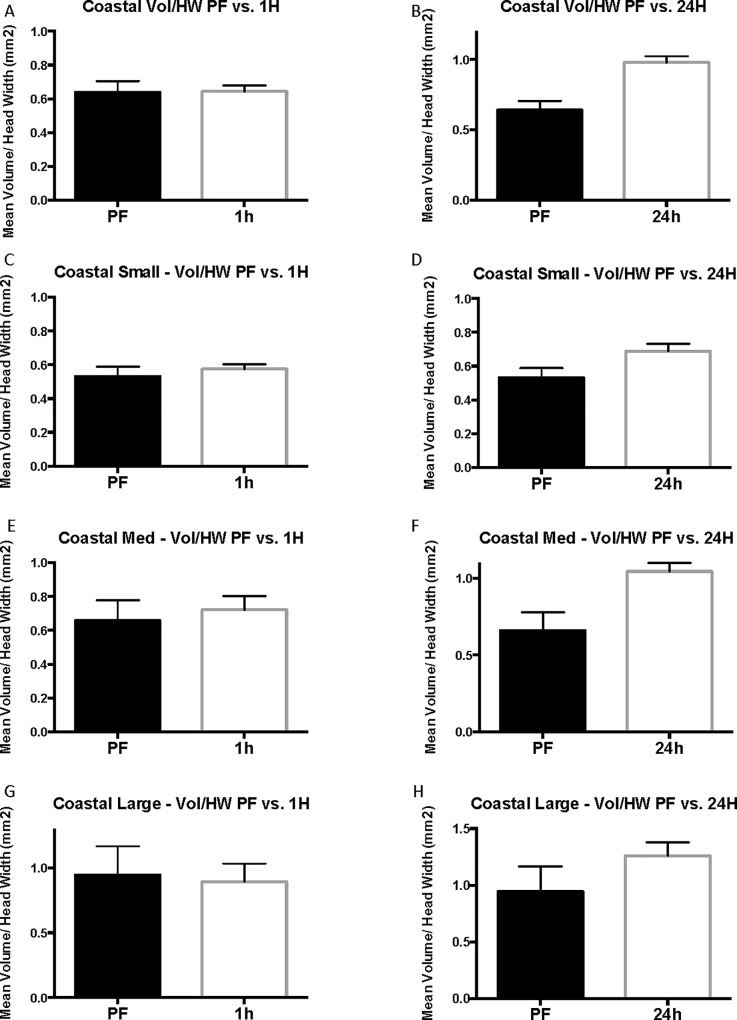
Size-standardized coastal results. Vol/HW refers to mean relative value that results from dividing venom sac volume by head width and is reported in mm^2^. The error bars represent standard error of the mean. PF refers to pre-flood cohorts, 1H refers to 1-hour cohorts, and 24h refers to 24-hour cohorts.

The results of the coastal worker size-standardized (relative value, r = venom sac volume/head width) analysis mirrored those presented above. As a whole, there was no significant change in the venom sac volumes of coastal workers from pre-flood (r = 0.64 ± 0.065 mm^2^) to 1-hour flood cohorts (r = 0.64 ± 0.033 mm^2^) (P = 0.6789, U = 884) (Fig 6A). There was, however, a decidedly significant increase in venom sac volume per unit head width over 24 hours, resulting in 24-hour workers with an average relative value of 0.98 ± 0.044 mm^2^ (P = 0.0001, U = 809.5) (Fig 6B) or 35% increase.

**Fig 7:**
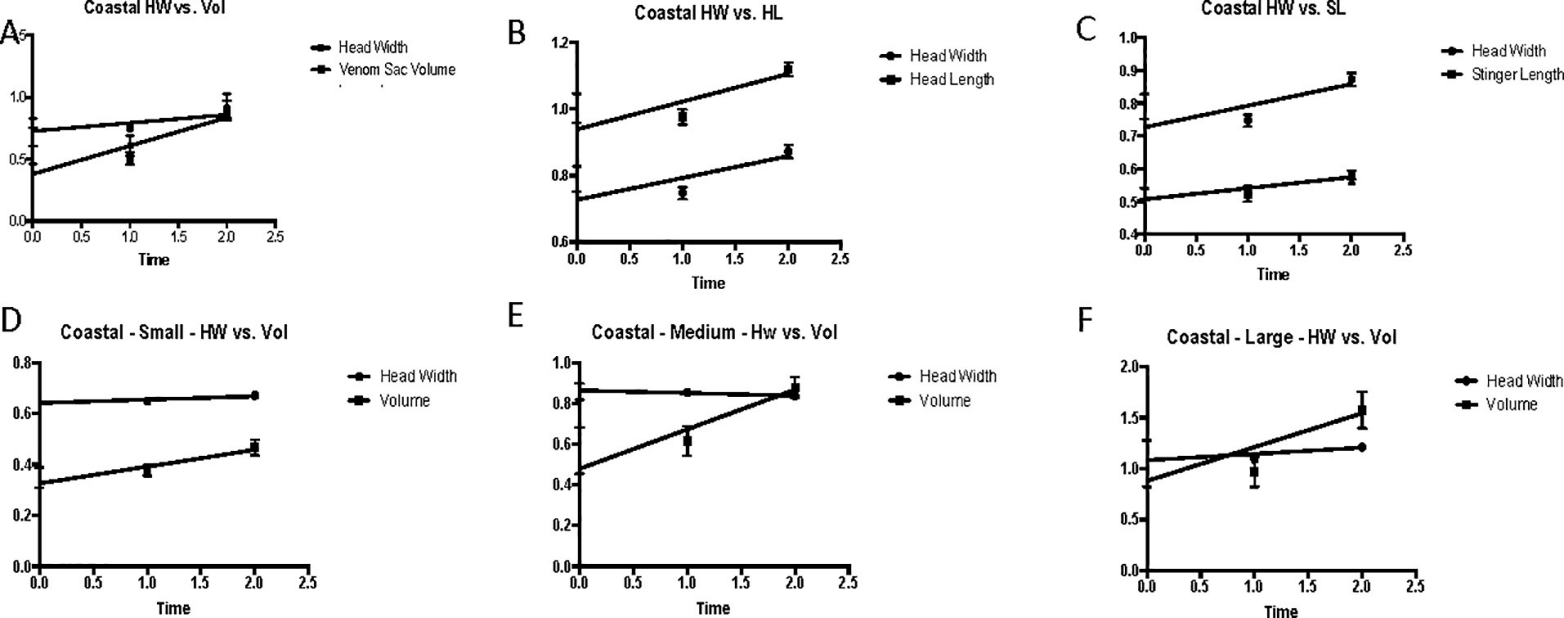
Linear regression analysis of coastal workers during the flood. A. Total coastal head width (HW) and volume (Vol) vs. time. B. Total coastal head width and head length vs. time. C. Total coastal head width and stinger length vs. time. D. Small coastal head width and volume vs. time. E. Medium coastal head width and volume vs. time. F. Large coastal head width and volume vs. time.

In agreement with the coastal results, small and medium workers faced an increase in venom sac volume per unit head width over a 24-hour flooding period. Small coastal pre-flood workers had a mean relative value of 0.53 ± 0.580 mm^2^, and the 24-hour cohort had a significantly higher mean relative value of 0.69 ± 0.043 mm^2^ (23%, corrected P = 0.146, t = 2.013, df = 52) (Fig 7D). Head widths in this size group range 0.57–0.74 mm whereas, medium workers ranged in head width from 0.75 to 0.99 mm thus possibly explaining the difference. Medium coastal workers experienced an increase in venom sac volume per unit head width unit from 0.66 ± 0.120 mm^2^ before flooding compared with 1.04 ± 0.055 mm^2^ 24 hours into flooding (37%, corrected P = 0.050, U = 63) (Fig 7F). As seen above, large coastal workers (head widths = 1.0–1.48 mm) experience no change in venom sac volume (Table 2).

**Table 2:** Fire ant size venom sac volumes (calculated) corrected for body size via head width and results of statistical analysis for all coastal ants standardized for size. Bonferroni corrections were applied to the p-values used in multiple comparisons to reduce the possibility of a Type-1 error. Those p-values which were corrected are noted with a • symbol. Corrected p-values were compared to the Bonferroni-corrected α_critical_ = 0.049.

Factor(s) tested	Test	Mean ± SEM	Range	N	P	Different	t	U	df
Total Coastal Pre-flood Vol/HW		0.64 ± 0.065	0.21–1.7	26					
Total Coastal 1-hour Vol/HW	Mann-Whitney	0.64 ± 0.033	0.26–1.74	72	0.999•	No		884	
Total Coastal 24-hour Vol/HW	Mann-Whitney	0.98 ± 0.044	0.21–3.28	118	0.0003•	Yes		809.5	
PF to 24h relative increase = 65%									
Small Coastal Pre-flood Vol/HW		0.53 ± 0.580	0.21–0.94	15					
Small Coastal 1-hour Vol/HW	Mann-Whitney	0.58 ± 0.029	0.30–1.13	46	0.999•	No		303	
Small Coastal 24-hour Vol/HW	Unpaired t- test	0.69 ± 0.043	0.21–1.43	39	0.146•	No	2.013		52
PF to 24h relative increase = 65%									
Medium Coastal Pre-flood Vol/HW		0.66 ± 0.120	0.41–1.09	6					
Medium Coastal 1-hour Vol/HW	Mann-Whitney	0.72 ± 0.082	0.26–1.74	19	0.999•	No		50	
Medium Coastal 24-hour Vol/HW	Mann-Whitney	1.04 ± 0.055	0.27–2.11	51	0.050•	No		63	
Large Coastal Pre-flood Vol/HW		0.94 ± 0.221	0.43–1.73	5					
Large Coastal 1-hour Vol/HW	Mann-Whitney	0.89 0.139	0.29–1.40	7	0.999•	No		16	
Large Coastal 24-hour Vol/HW	Mann-Whitney	1.26 ± 0.118	0.42–0.32	28	0.959•	No		50	

Size-standardized coastal unpaired t-tests and Mann-Whitney tests. Vol/HW refers to mean relative value that results from dividing venom sac volume by head width and is reported in mm^2^. N represents the number of workers within the corresponding group, P is the p-value, and U is U-value from Mann-Whitney U tests. Tests that determined significant (p < 0.005) differences are marked by the word “yes” under the column labeled “different.”

Results of linear regression analyses for total (all groups treated together), small, medium, and large coastal groups are listed in the S4 Table, and the corresponding graphs are depicted in Fig 7. Linear regression of all coastal venom sac volume data suggests that during the flooding, venom sac increases by the equation Y = 0.23X + 0.38, where the slope is significant (P <0.001, R^2^ = 0.06032, F = 15.77). Venom sac volume increases in small workers and is described by Y = 0.07X + 0.33 where the slope is significant (P = 0.007, R^2^ = 0.07193, F = 7.596) but not as steep as the slope which describes increase in volume in medium workers (Y = 0.19X + 0.48, P = 0.0028, R^2^ = 0.1145, F = 9.572). The slope describing the change in venom sac volumes of large workers was not significantly nonzero (P = 0.0875).

### Inland results (Experiment 3)

**Fig 8:**
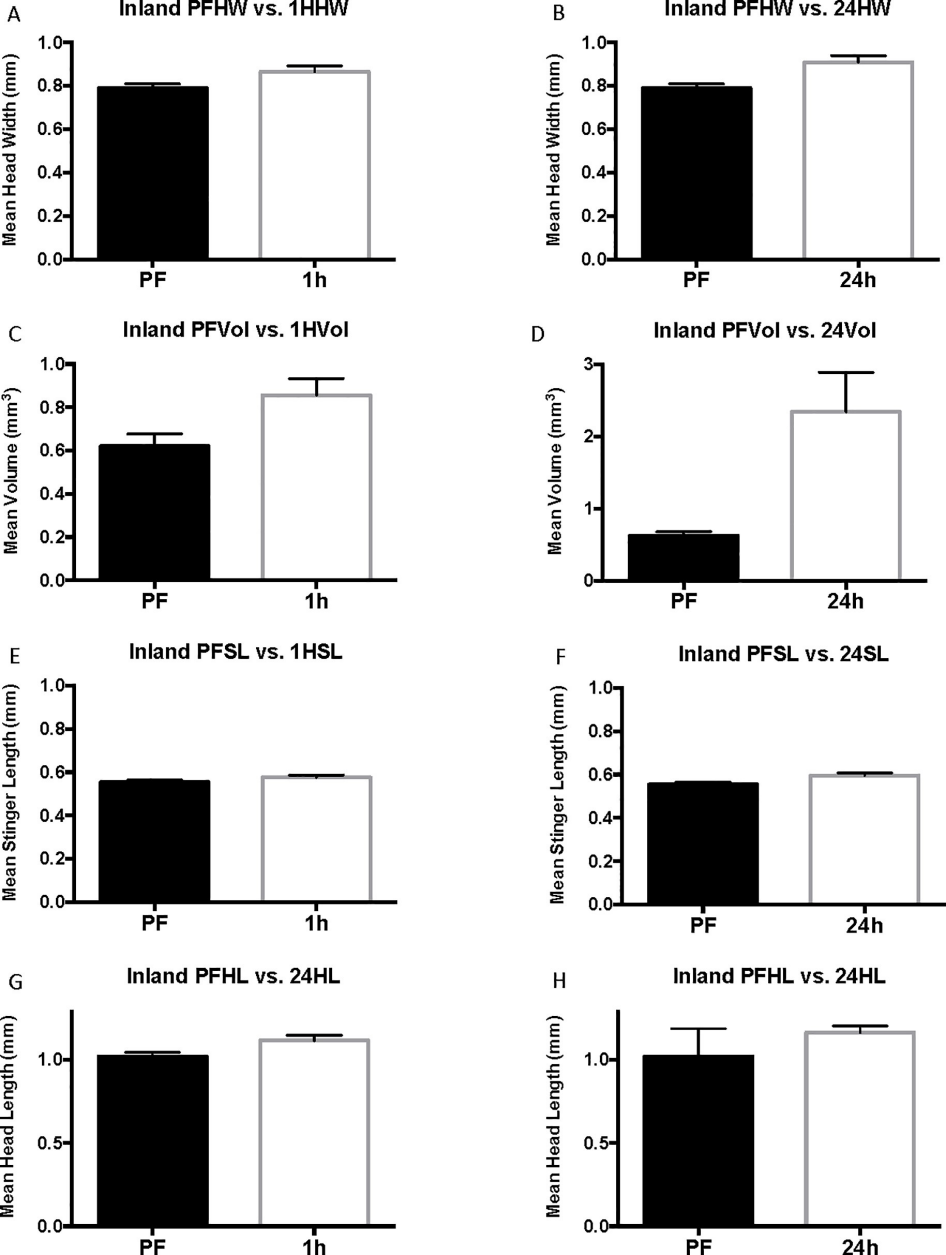
Complete inland results. Mean head width, mean stinger length, and mean head length are reported in mm. Mean volume is in mm^3^. The error bars represent standard error of the mean. PF refers to pre-flood cohorts, 1H refers to 1-hour cohorts, and 24h refers to 24-hour cohorts.

In inland colonies, there is a general increase in body size as indicated by head width compared with coastal colonies. Individual colony results are presented in S1–S8 Tables because although multiple ants were tested among each colony, the colony itself is considered the replicate in this experiment. The individual ants are considered subsamples. Comparable to coastal colonies, 24-hour inland cohorts were larger than pre-flood cohorts in head width, head length, and stinger length (Fig 8). Before flooding, inland ants had a mean head width of 0.79 ± 0.020 mm whereas workers taken 24-hours into flooding had a much larger average head width of 0.91 ± 0.031 mm (13%, P = 0.0102, U = 860) (Fig 8B). Head length similarly was smaller in 1.02 ± 0.024 pre-flood compared with 1.16 ± 0.038 24 hours following commencement of flooding (12%, Fig 8H). The sharp, pointed stingers of the 24-hour cohort were longer (0.60 ± 0.012 mm) than stingers of the pre-flood cohort (0.55 ± 0.010 mm, P = 0.0125, U = 870) (-9%, Fig 8F). Inland results are similar to those of coastal ants with the exception that venom sac enlargement was observed in 1-hour cohorts (28%) in addition to 24-hour cohorts in inland ants. Venom sac volume increased from 0.620 ± 0.057 mm^3^ before flooding, to 0.86 ± 0.076 mm^3^ 1 hour into the flood (28%, corrected P = 0.075, U = 905.5), to 1.01 ± 0.096 mm^3^ 24 hours into the flood (39%, corrected P = 0.0093, U = 806) (Fig 8C and 8D). Additionally, the mean venom sac volume of inland ants is greater than that of coastal ants for each of the flood cohorts. (S1 and S4 Tables).

**Fig 9:**
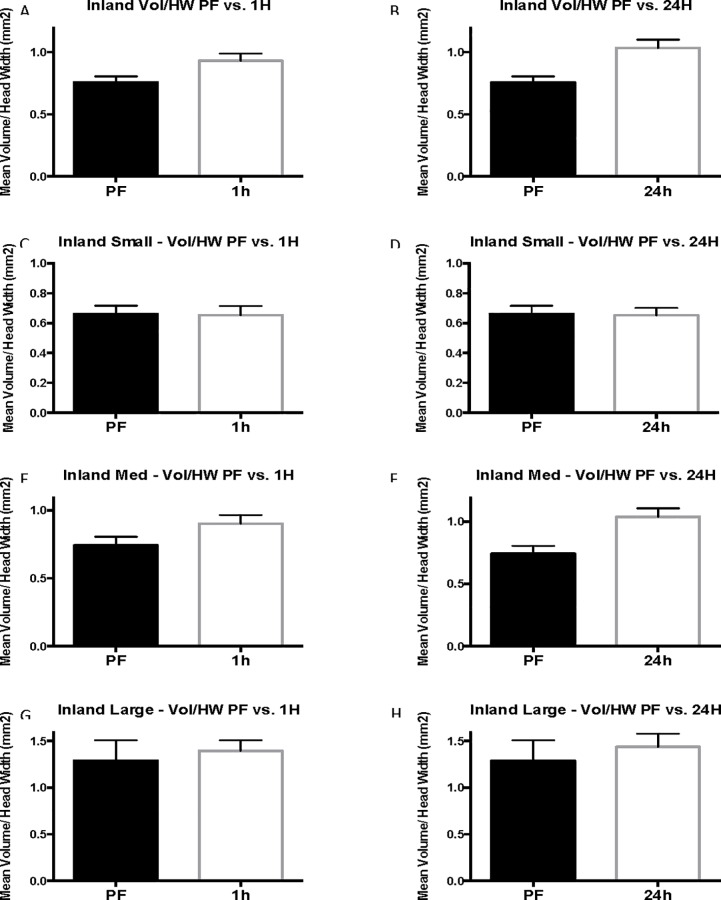
Size-standardized inland results. Vol/HW refers to mean relative value that results from dividing venom sac volume by head width and is reported in mm^2^. The error bars represent standard error of the mean. PF refers to pre-flood cohorts, 1H refers to 1-hour cohorts, and 24h refers to 24-hour cohorts.

**Fig 10:**
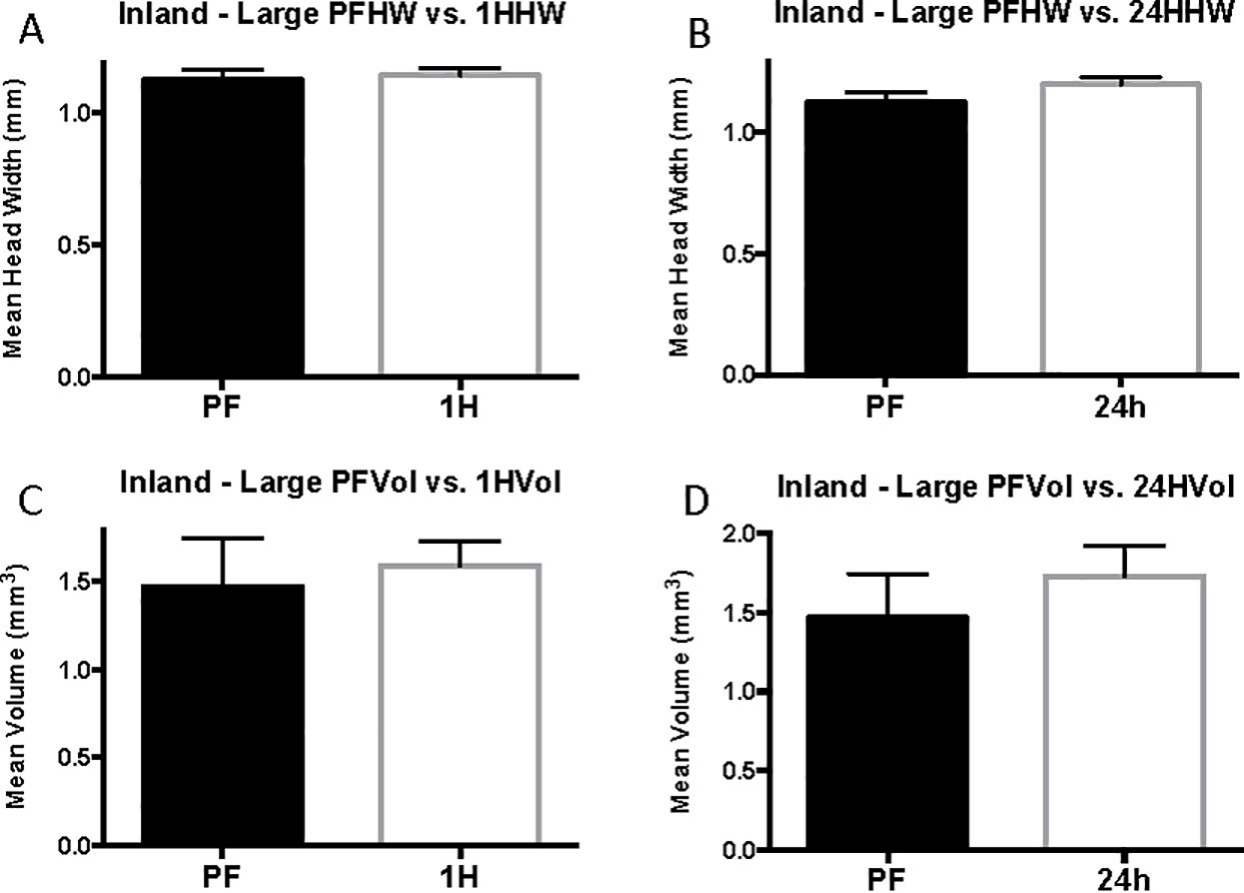
Small inland head with and venom sac volume. Mean head width is in mm. Mean venom sac volume is in mm^3^. The error bars represent standard error of the mean. PF refers to the pre-flood cohort (n = 25), 1H refers to the 1-hour cohort (n = 18), and 24h refers to the 24-hour cohort (n = 16).

**Fig 11:**
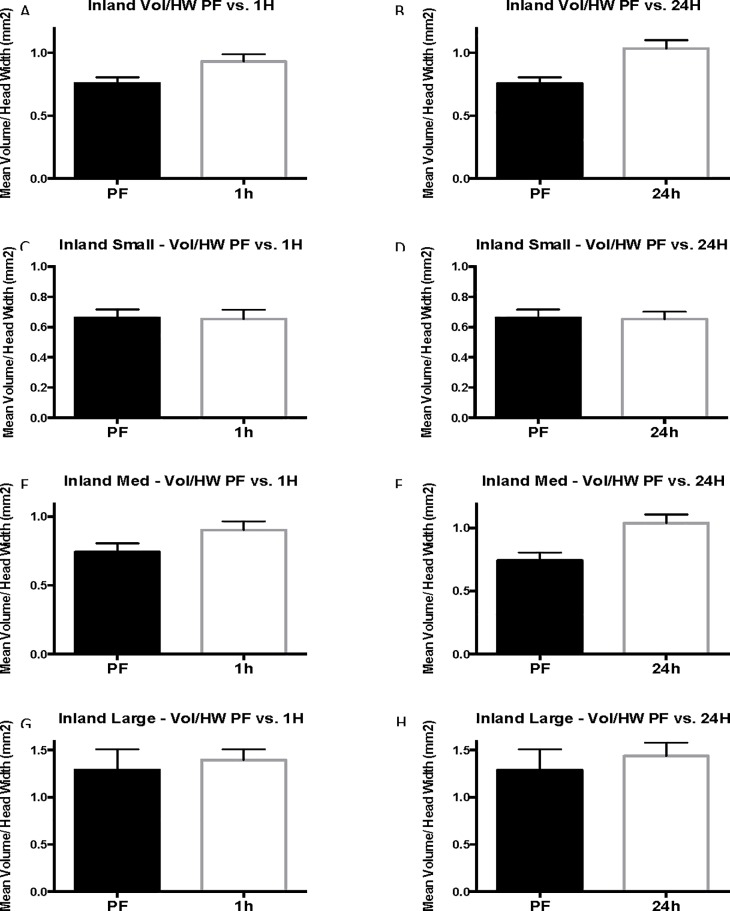
Large inland head with and venom sac volume. Mean head width is in mm. Mean venom sac volume is in mm^3^. The error bars represent standard error of the mean. PF refers to the pre-flood cohort (n = 5), 1H refers to the 1-hour cohort (n = 12), and 24h refers to the 24-hour cohort (n = 15).

As with coastal colonies, grouping of the inland worker data by head width size controls for variation in head width across cohorts and accounts for differences in head width for each size group. The mean head width for pre-flood, 1-hour (corrected P = 0.9999, U = 187), and 24-hour cohorts is statistically the same (corrected P = 0.9999, U = 171 (S6 Table). When inland cohorts are grouped by head width, venom sac enlargement is seen in 1-hour and 24-hour cohorts of medium workers (head width 0.75 mm ≤ 1 mm, range = 0.63–0.74, N = 59) (Fig 9). Unpaired t-test determined that the increase from 0.60 ± 0.050 mm^3^ in pre-flood medium workers to 0.78 ± 0.064 mm^3^ in 1-hour medium workers is significant (23%, corrected P = 0.0993, t = 2.213, df = 37). The venom sac volume (0.90 ± 0.07 mm^3^) of 24-hour medium workers was also significantly larger than that of the pre-flood cohort (33%, corrected P = 0.0036, t = 3.525, df = 36). The 1-hour and 24-hour cohorts of small and large workers were not different from pre-flood cohorts in any of the measured parameters (S6 Table, Figs 10 and 11).

The results of the inland worker size-standardized (relative value, r = venom sac volume/head width) analysis were similar to the results discussed above with the exception that there was no change in venom sac volume per unit head width after 1 hour for medium workers (corrected P = 0.0084). There was, however, a significant increase after 24 hours, and 24-hour workers had a mean venom sac volume of 1.04 ± 0.068 mm^3^ per mm head width compared with just 0.74 ± 0.062 mm^3^ per mm before flooding (29%, Fig 9E and 9F). In line with the inland results, small and large workers experienced no change in venom sac volume per unit head width. Small inland workers (head width = 0.63–0.74 mm) had a 24-hour relative value of 0.65 ±0.049 mm^2^ (P = 0.6965), and large inland workers had (head width = 1.05–1.37 mm) had a much higher mean relative value of 1.44 ± 0.1417 mm^2^ (55%, P = 0.9999).

**Fig 12:**
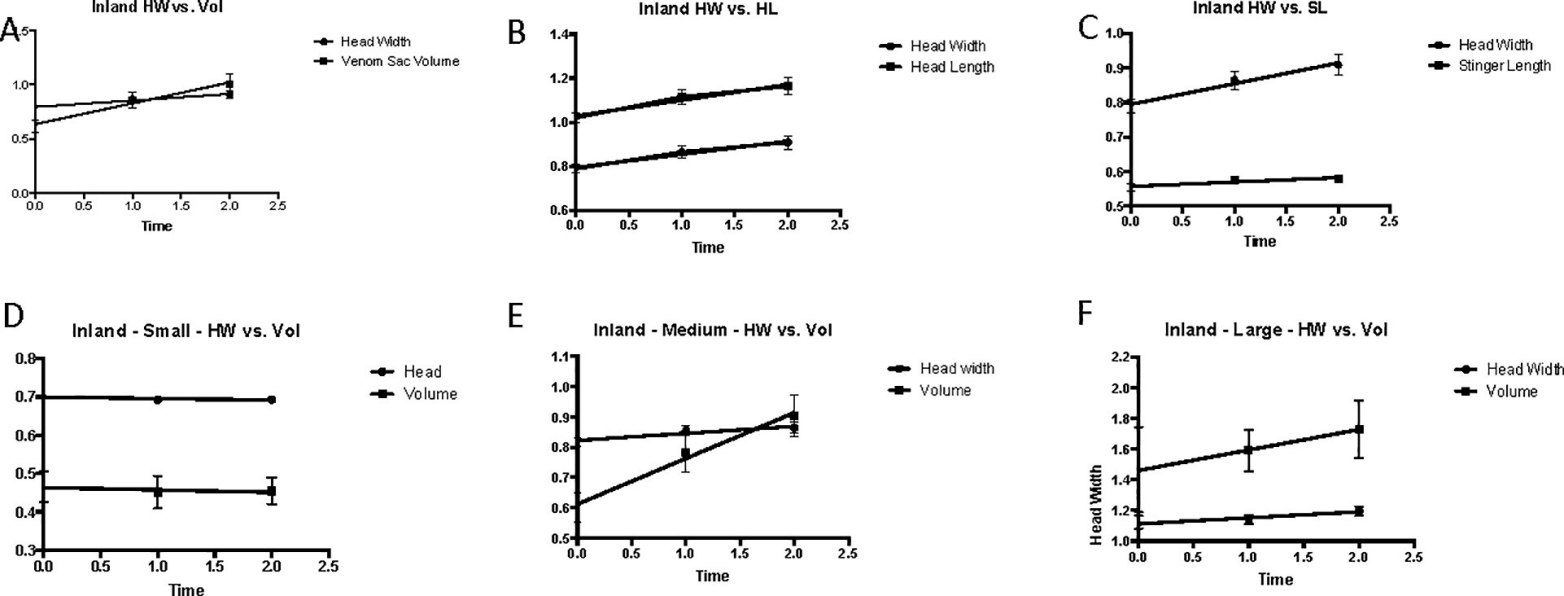
Linear regression analysis of inland workers during the flood. A. Total inland head width and volume vs. time. B. Complete inland head width and head length vs. time. C. Complete inland head width and stinger length vs. time. D. Small inland head width and volume vs. time. E. Medium inland head width and volume vs. time. F. Large inland head width and volume vs. time.

Results of linear regression analyses for complete, small, medium, and large inland groups are listed in (S8 Table), and the corresponding graphs are depicted in Fig 12. Linear regression of inland venom sac volume data suggests that during flooding, venom sac increases by the equation Y = 0.19X + 0.64, where the slope is significant (P = 0.0006, R^2^ = 0.07772, F = 12.39). Of the size groups, only medium workers had a significantly non-zero slope showing an increase in venom sac volume over the length of the flood event (Y = 0.15X + 0.61, P = 0.0011, R^2^ = 0.1754, F = 11.91). The slope describing the change in venom sac volumes of small inland workers was determined not to be significantly nonzero (P = 0.8294). The same was true for large inland workers (P = 0.3815).

The increase in venom sac volume of small workers is described by Y = 0.07X + 0.33 where the slope is significant (P = 0.007, R^2^ = 0.07193, F = 7.596) but not as steep as the slope which describes an increase in medium workers (Y = 0.19X + 0.48, P = 9.572, R^2^ = 0.1145, F = 9.572). The slope describing the change in venom sac volumes of large workers was determined not to be significantly nonzero (P = 0.0875).  

## Discussion

Regardless of salinity of the floodwater, flooding ants from the bottom up and by dripping with an IV bag simulating rain more closely resembles what happens in nature during rain events and storm surge or water stacked up by offshore winds and other ocean oscillations. Alternatively, the Banks et al. (1981) protocol and Chen (2007) suggest pouring water from above (~1 L at a time), and their technique requires poking and stirring the soil with a stick to remove pockets of brood left behind by the workers as the water rises [22,24]. Our new technique does not require any poking to release the brood as it allows the workers to use their natural behaviors to move the eggs, brood, and queens, to higher ground ensuring most, if not all of the offspring and queens do not become trapped. Similar to Adams et al. [13], late stage (instar) brood trapped air bubbles next to their bodies with hooked hairs; this air bubble was used as a pontoon upon which the adult ants rafted. The ants flooded with saltwater rafted similarly to Adams et al. [13] with the brood as the basis for the raft; however, within a day, this raft base structure changed to dead ants that were semi-buoyant contrary to Adams et al. [13].

Flooding the colonies from the bottom simulated a natural rafting situation permitting the ants enough time to remove the majority of the brood from the soil and allowed the researchers to have better control in attaining an ant raft. Since the water rose slowly from the bottom of the chamber and was not crashing into the soil from above smashing their nest construction and locally drowning the workers, the workers had time to gather brood and bring them to higher ground to escape the threat of the rising water. The stick added to the center of the flood chamber to keep the colony from escaping is not out of the realm of what could happen in a natural setting (e.g., a plant). In nature, rafting colonies anchor to anything within reach in order to survive flooding [13]. In addition to the brood, dead ants and grass pieces were used to raft, and control colonies had extra support from a layer of plaque growing on the surface of the water. This plaque layer provided enough surface tension for ants to cling and climb on top of it suggesting that in stagnant water conditions, this may be an alternative means of rafting and survival for ants.

One of the main differences between the size grouping results of coastal and inland colonies is that small coastal colonies exhibited a 25% increase in venom volume (24h) whereas inland colonies venom volumes did not change. Small inland ants may not play as large a role in colony defense as small coastal ants. This potential change in role of small coastal ants may help explain the perception that coastal ants are more aggressive even though the colony sizes appear to be similar. Further, there was no difference between inland and coastal ants’ venom sac volumes for medium-sized ants. However, flooded coastal ants’ venom sac volumes increased more than inland ants’ volumes despite starting out smaller. Both inland and coastal colonies experienced venom sac enlargement after 24 hours. Venom sac volumes of coastal ants increased 72% whereas inland ants venom sacs of inland ants increased 34% indicating possible phenotypic changes in coastal ants in relation to the stress of inundation with salt water.

When examined under a microscope, brood in direct contact with the saltwater appeared shriveled, but brood on top of the bottom layer had the appearance of non-shriveled brood under non-saltwater circumstances [13]. This result indicates the possibility that brood not in contact with salt water, were still viable at day one. Saltwater and brackish environments present considerable challenges to insect survival. Those with an inability to fly to a more suitable location must find ways to adapt. Our results indicate that fire ants have adapted behaviorally and physiologically to the threat of rising water. Further, this adaptation may be useful in warding off threats from predators while the ants are exposed as noted by Haight 2006 [20].

In both inland and coastal *S*. *invicta* colonies, 24-hour cohorts were made up of individuals that were generally larger than pre-flood cohorts as measured by head width. Head width is an excellent parameter to estimate differences in body size of fire ants [26–28]. The larger head width size was accompanied by larger head lengths, stinger lengths, and subsequently higher venom sac volume for both inland and coastal colonies, which follows Wood and Tschinkel (1981) and Kaspari and Wieser (1999) [26–28]. Three of the four coastal colonies were shown to individually experience the success of larger ants when flooded as shown by larger head widths during flooding (Fig 2).

The larger head width sizes during flooding were neither seen by Papillion et al. (2011) [21] nor were changes in venom sac length or stinger length. Although the greater size of stinger length after 1 and 24 hours of flooding was deemed not significant, it was found to be described by the equation flooded stinger length = 0.190 + (0.437*non-flooded head width) (Papillion et al. 2011) [21]. Regression analysis of coastal and inland colonies in this study indicates a linear relationship between stinger length and head width, and both are larger in ants that have experienced flooding (Figs 7 and 12, S2 and S6 Tables). Papillion et al. (2011) found venom sac width of flooded ants to be related to head width by the equation flooded venom sac width = 0.0785 + (0.351*flooded head width) [21]. Although the change in venom sac width and venom sac length were not analyzed separately, the results of this study support an increase in one of both parameters as venom sac volume increases significantly during flooding. Linear regression for venom sac volume was significant for inland workers as well as coastal workers (Figs 7 and 12, S2 and S6 Tables). The larger variation in body sizes measured by this study may be because of the large geographic range of collection (three parishes) rather than one location–East Baton Rouge Parish.

Papillion et al. (2011) observed a 79% increase in venom sac width in inland ants after 1 hour of flooding, but no subsequent increase after 24 hours [21]. This result is particularly interesting in light of the differences in venom sac enlargement in inland and coastal workers. As a whole, inland workers venom sac enlargement from 0.620 ± 0.057 mm^3^ to 0.86 ± 0.076 mm^3^ or 28% increase after one hour of flooding (P = 0.025, U = 905.5) and individual colony venom sac enlargement is seen in colonies I1 and I3 (S4 Fig, Table 1). No coastal colonies experienced a change in venom sac volume after only one hour, suggesting a difference in either venom production or taking in of water in the two groups and suggesting that they are adapted to the stress of tidal flooding but not prolonged flooding. Papillion et al.’s (2011) results differ [21] from those of this study in that inland workers also experienced venom sac enlargement after 24 hours [21]. Whereas, inland ants as a whole saw an increase in venom sac volume after 1 hour and again after 24 hours (Fig 11C and 11D). Looking at individual inland colonies, however, only colony I3 exhibited this trend (see SI). Venom sac volume increased from 0.76 ± 0.127 mm^3^ to 1.23 ± 0.169 mm^3^ (P = 0.0435, t = 2.181, df = 17) after one hour for an increase of 38% then to 1.57 ± 0.263 mm^3^ (P = 0.016, t = 2.673, df = 17) after 24 hours for an increase of 52% (S4 Fig, Table 1). On the other hand, Colony I1 experienced an increase in venom sac volume from 0.52 ± 0.095 mm^3^ to 0.93 ± 0.114 mm^3^ after 1 hour (44% increase, P = 0.0034, U = 94) but no significant change after 24 hours, conforming to Papillion et al.’s (2011) results [21]. It is not known how long the increase in venom volume lasts. The results of this study suggest that larger workers either survive floods better or have more prominent roles in rafting, which led to a greater representation of larger workers in 1-hour and 24-hour cohorts. It is doubtful that the head width of individual ants actually increases during flooding due to the sclerotized nature of the exoskeleton; what is proposed is that these larger ants are either more numerous and/or more accessible during flooding and rafting. Studies have shown that larger workers have lower metabolic rates and live longer than small workers [25, 27, 29–30]. The greater longevity seen in larger workers may explain their ability to survive floods better. Larger workers represent a considerable investment of nutrients by the colony.

Grouping by head-width size controls for worker size and provides a look at different worker classes across stages of the flood event. As size polyethism is a foundational aspect of fire ant social structure [31], differences in venom production or content may provide insight into roles of worker of different sizes in defense. In both inland and coastal colonies [32], medium workers make up a higher percentage of the 24-hour cohort than small or large workers. Medium workers represent 38.00% of inland and 42.86% of coastal ants collected after 24 hours of flooding. Large workers underwent the most significant increase in representation during flooding from only 10.20% to 30.00% in inland colonies and 19.23% to 38.89% in coastal colonies. Small workers went from representing over 50% of non-flooded cohorts to only 32% of the 24-hour inland cohort and 32.77% of 24-hour coastal cohort indicating they may not fare well during flooding. In addition to being present in larger percentages in flooded colonies, both inland and coastal medium workers experienced the greatest increase in venom sac volume during flooding.

In inland colonies, medium workers experienced a volume increase from 0.60 ± 0.050 mm^3^ to 0.90 ± 0.07 mm^3^ over 24 hours of flooding or a 33% increase, which corresponds to an increase in venom sac volume per millimeter head width from 0.74 ± 0.062 mm^3^ per mm to 1.04 ± 0.068 mm^3^ (29% increase). In coastal colonies, medium workers experienced a volume increase from 0.57 ± 0.115 mm^3^ to 0.88 ± 0.051 mm^3^ or 35% increase during 24 hours of flooding, which corresponds to an increase in venom sac volume per millimeter head width from 0.66 ± 0.120 mm^2^ before flooding to 1.04 ± 0.055 mm^2^ or 37% increase. Small coastal ants experienced a smaller increase in venom sac volume per unit head width from 0.53 ± 0.580 mm^2^ to 0.69 ± 0.043 mm^2^ representing a 23% increase. The increase in small worker venom sac volume in coastal colonies suggests that small workers may have an adjusted role in coastal colonies. Perhaps small coastal workers are more involved in defense of the colony, which is at greater risk of flooding than inland colonies.

Typically, younger workers tend to brood deep within the colony while older workers forage [10, 16, 27], but size and age are not linked in ants. Deslippe and Guo (2000) found that intermediate-age workers produce more venom than young and old workers [18]. If the age of colony correlates strongly to overall increased sizes of ants within a colony, this observation may be applicable to medium-sized workers, which experience a more significant increase in venom sac volume than smaller and larger workers. Deslippe and Guo (2000) also found that venom composition changes with age to include a higher percentage of unsaturated alkaloids [18]. Further analysis of venom alkaloids by worker size may provide more information about the roles of workers in colony defense over the lifespan. Additionally, venom constituents of flooded ants need to be examined.

A note on the experimental design in retrospect: the authors noticed while writing this manuscript that when the experiment was performed, one additional set of samples should have been taken immediately after the colonies were brought into the lab. Appropriate samples were taken before the "treatment," and correctly during treatment, and the results are comparable to our past experiments and each other. In future experiments, venom volumes should be measured before any colonies are flooded as the researchers may have “selected” for ants that survived flooding prior to conducting the experiment.

There is a definite increase in venom sac volume during flooding, but the mechanism of venom sac enlargement remains to be elucidated. Vander Meer and Alonso (2002) demonstrated that the queen pheromones control venom production in workers, but the mechanism during flooding is unknown [33]. Papillion et al. (2011) offered three explanations for the 79% increase in venom sac volume observed in inland *S*. *invicta* workers [21]: (1) ants are taking up water from the environment, (2) venom production is stimulated as a defense mechanism, or (3) taking up water from the environments stimulates a mechanism which results in increased venom production. It is possible that flooded ants take in water by diffusion, leading to dilution of venom and increased venom sac volume. If this is the case, it may explain the differences in venom sac enlargement seen between inland and coastal colonies. Inland colonies experienced increases in venom sac volume after one hour of flooding, while coastal ants did not. A slower rate of diffusion of 10 ppt saltwater compared to freshwater may explain these results as an adaptation on the part of the ants to the stress of rising tides. According to Haight (2006), pre-flood and flooded venom blots had the same luminance values. Therefore, it is unlikely that the enlargement is due to diffusion as diluted venom would have a lesser luminance value [20]. Haight (2006) suggests that the concentration of alarm pheromones in rafting ants triggers a heightened defensive behavior [20]. This explanation is a better fit considering that venom sac volume changes during flooding differ by size of the ant. If the method of venom sac enlargement was simple diffusion, ants of different size should retain the same water uptake ability. However, *S*. *invicta* responds to pheromones differently based on context and caste. Thus, high concentrations of alarm pheromones may act differently on small and large workers, resulting in an increase in venom sac volume during rafting. Further investigation of pheromone communication among colony members may provide more insight into the roles of different sized workers in rafting and colony defense.

Other species have experienced morphological changes in the aftermath of stressors. Hurricanes Irma and Maria appear to have selected for changes in body and toe pad size and limb length in *Anolis scriptus* on the Turks and Caicos archipelago [34]. This has also been seen in beetle populations in areas disturbed by hurricanes [35].

Fire ants can and will raft on high salinity water. This behavior represents an adaptation of coastal ants to the stress of sea-level rise. Furthermore, sea-level rise may cause stings by flooded ants to be more severe because of increased venom volume and larger worker body size. Larger sized ants have more venom and smaller coastal fire ants may be more involved in colony defense. A proximate change was measured in the ants’ behavioral and physiological response to flooding. Then, the ultimate ecological changes in the face of rising seas can be predicted with the fire ant rafting information and the venom volume shown in this research.

## Supporting information

S1 Fig Individual coastal colony head widths. Mean head width (abbreviated HW) is reported in mm. The error bars represent standard error of the mean. PF refers to the pre-flood cohort that was taken immediately before flooding. 1-h refers to the cohort taken 1 hour into flooding and 24 h refers to the cohort taken 24 hours into flooding.
(TIFF)

S2 Fig Individual inland colony head widths. Mean head width (abbreviated HW) is reported in mm. The error bars represent standard error of the mean. PF refers to pre-flood cohorts, 1H refers to 1-hour cohorts, and 24h refers to 24-hour cohorts.
(TIFF)

S3 Fig Individual coastal colony venom sac volumes. Mean volume (abbreviated Vol) is reported in mm^3^. The error bars represent standard error of the mean. PF refers to pre-flood cohorts, 1H refers to 1-hour cohorts, and 24h refers to 24-hour cohorts.
(TIFF)

S4 Fig Individual inland colony venom sac volumes. Mean volume (abbreviated Vol) is reported in mm^3^. The error bars represent standard error of the mean. PF refers to pre-flood cohorts, 1H refers to 1-hour cohorts, and 24h refers to 24-hour cohorts.
(TIFF)

S1 Table Individual colony unpaired t-tests and Mann-Whitney tests. Head width, stinger length, and head length are reported in mm. Volume is in mm^3^. N represents the number of workers within the corresponding group, P is the p-value, t is the t-statistic from unpaired t-tests, U is U-value from Mann-Whitney U tests, and df is the degrees of freedom. Tests that determined significant (p < 0.005) differences are marked by the word “yes” under the column labeled “different”.
(PDF)

S2 Table Size-grouped coastal unpaired t-tests and Mann-Whitney tests. Head width, stinger length, and head length are reported in mm. Volume is in mm^3^. N represents the number of workers within the corresponding group, P is the p-value, and U is U-value from Mann-Whitney U tests. Tests that determined significant (p < 0.005) differences are marked by the word “yes” under the column labeled “different”.
(PDF)

S3 Table Size-standardized coastal unpaired t-tests and Mann-Whitney tests. Vol/HW refers to mean relative value that results from dividing venom sac volume by head width and is reported in mm^2^. N represents the number of workers within the corresponding group, P is the p-value, and U is U-value from Mann-Whitney U tests. Tests that determined significant (p < 0.005) differences are marked by the word “yes” under the column labeled “different”.
(PDF)

S4 Table Linear regressions of coastal workers during the flood. Equations are the form Y = mx +b where m is the slope and b is the y-intercept. R2 refers to the R–squared value, F refers to the F, and P is the p-value, which determines whether the slope is significantly non-zero.
(PDF)

S5 Table Complete inland unpaired t-tests and Mann-Whitney tests. Head width, stinger length, and head length are reported in mm. Volume is in mm3. N represents the number of workers within the corresponding group, P is the p-value, and U is U-value from Mann-Whitney U tests. Tests that determined significant (p < 0.005) differences are marked by the word “yes” under the column labeled “different”.
(PDF)

S6 Table Size-grouped inland unpaired t-tests and Mann-Whitney tests. Head width, stinger length, and head length are reported in mm. Volume is in mm^3^. N represents the number of workers within the corresponding group, P is the p-value, and U is U-value from Mann-Whitney U tests. Tests that determined significant (p < 0.005) differences are marked by the word “yes” under the column labeled “different”.
(PDF)

S7 Table Size-grouped inland unpaired t tests and Mann-Whitney tests. Head width, stinger length, and head length are reported in mm. Volume is in mm3. N represents the number of workers within the corresponding group, P is the p-value, and U is U-value from Mann-Whitney U tests. Tests that determined significant (p < 0.005) differences are marked by the word “yes” under the column labeled “different”.
(PDF)

S8 Table Size-standardized inland unpaired t-tests and Mann-Whitney tests. Vol/HW refers to mean relative value that results from dividing venom sac volume by head width and is reported in mm^2^. N represents the number of workers within the corresponding group, P is the p-value, and U is U-value from Mann-Whitney U tests. Tests that determined significant (p < 0.005) differences are marked by the word “yes” under the column labeled “different”.
(PDF)
