# Supplementary figures and images for: Effects of sea-level rise on physiological ecology of populations of a ground-dwelling ant
Source: PLoS One. 2020 Apr 17;15(4):e0223304. doi: 10.1371/journal.pone.0223304 (PMC7164625; doi:10.1371/journal.pone.0223304)

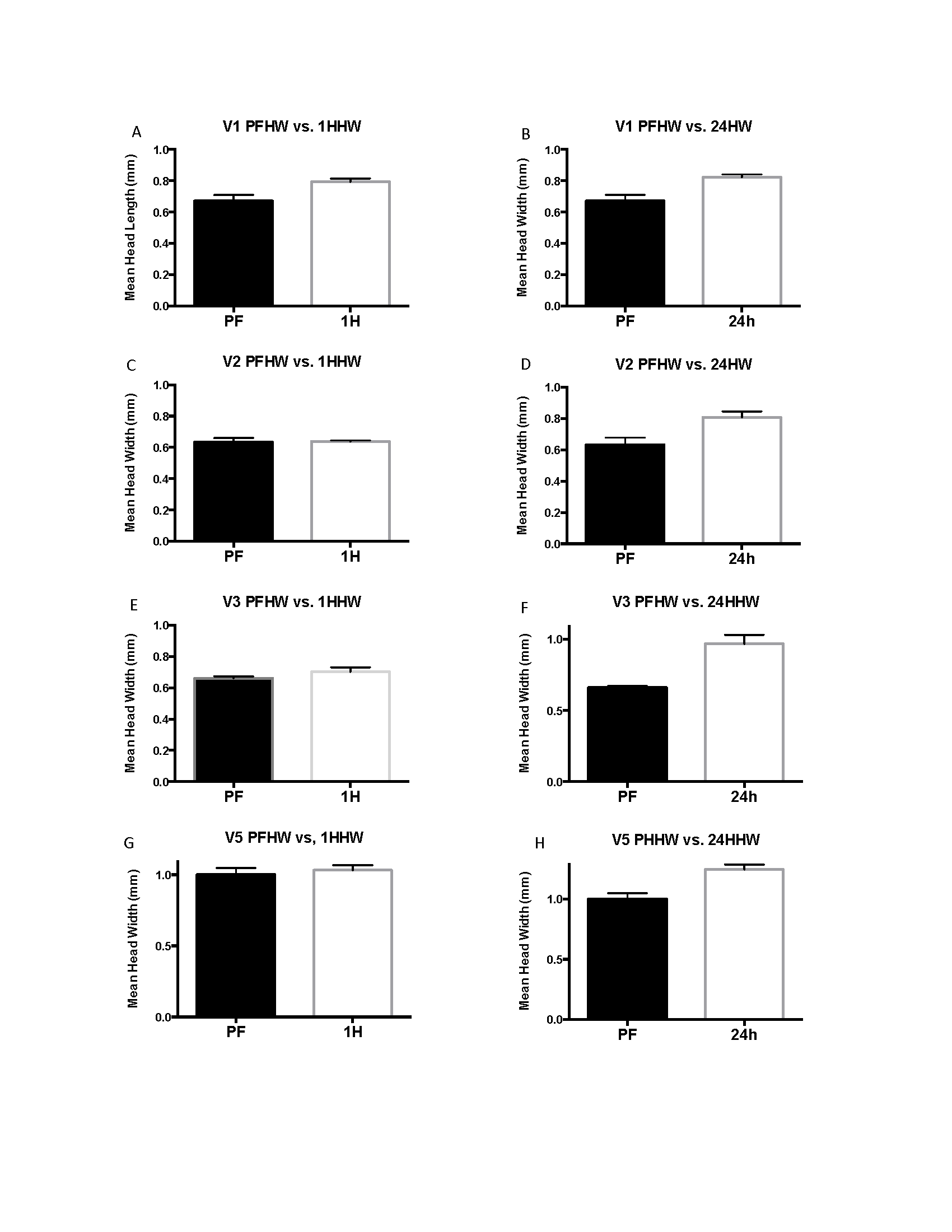

Supplement: S1 Fig — Mean head width (abbreviated HW) is reported in mm. The error bars represent standard error of the mean. PF refers to the pre-flood cohort that was taken immediately before flooding. 1-h refers to the cohort taken 1 hour into flooding and 24 h refers to the cohort taken 24 hours into flooding. (TIFF) [file pone.0223304.s001.tiff]

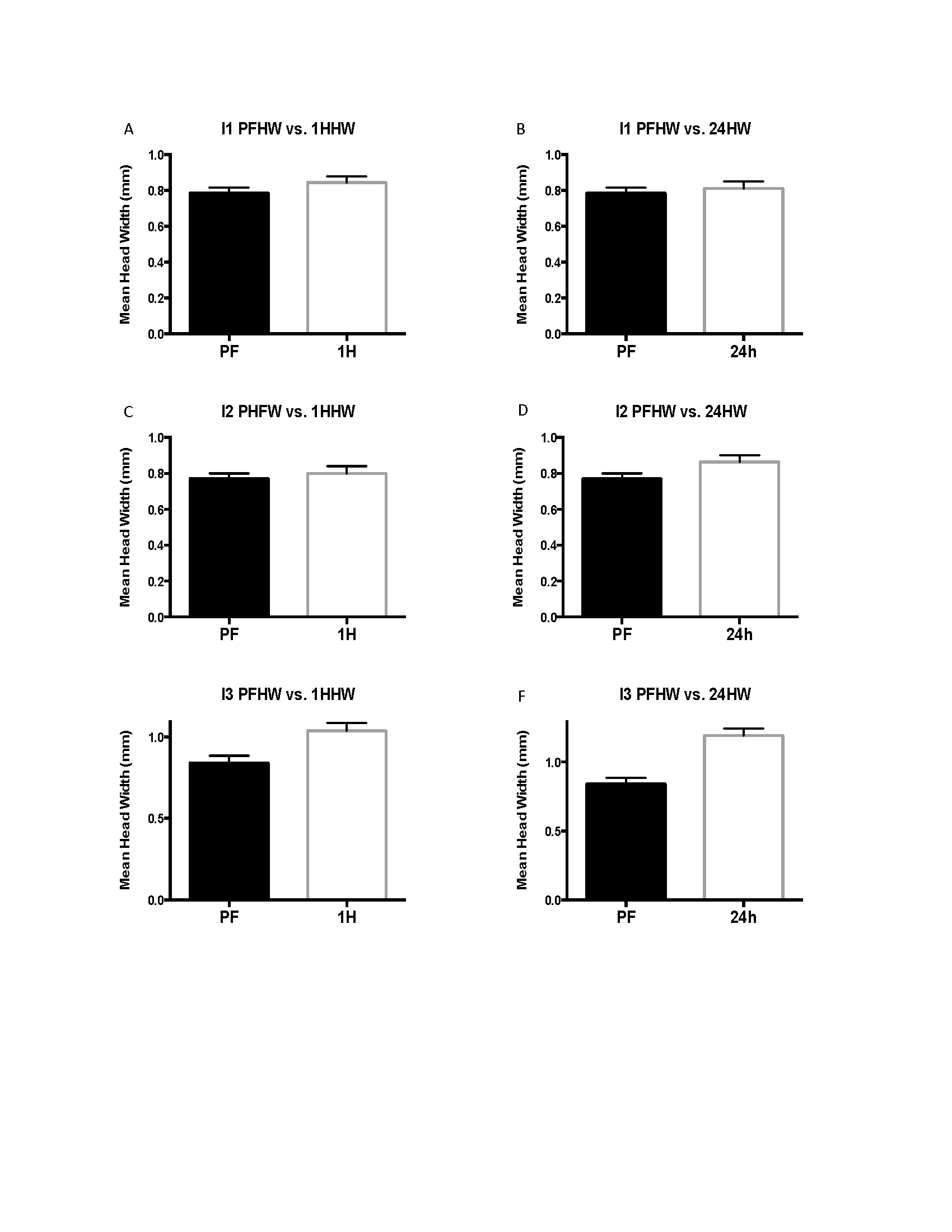

Supplement: S2 Fig — Mean head width (abbreviated HW) is reported in mm. The error bars represent standard error of the mean. PF refers to pre-flood cohorts, 1H refers to 1-hour cohorts, and 24h refers to 24-hour cohorts. (TIFF) [file pone.0223304.s002.tiff]

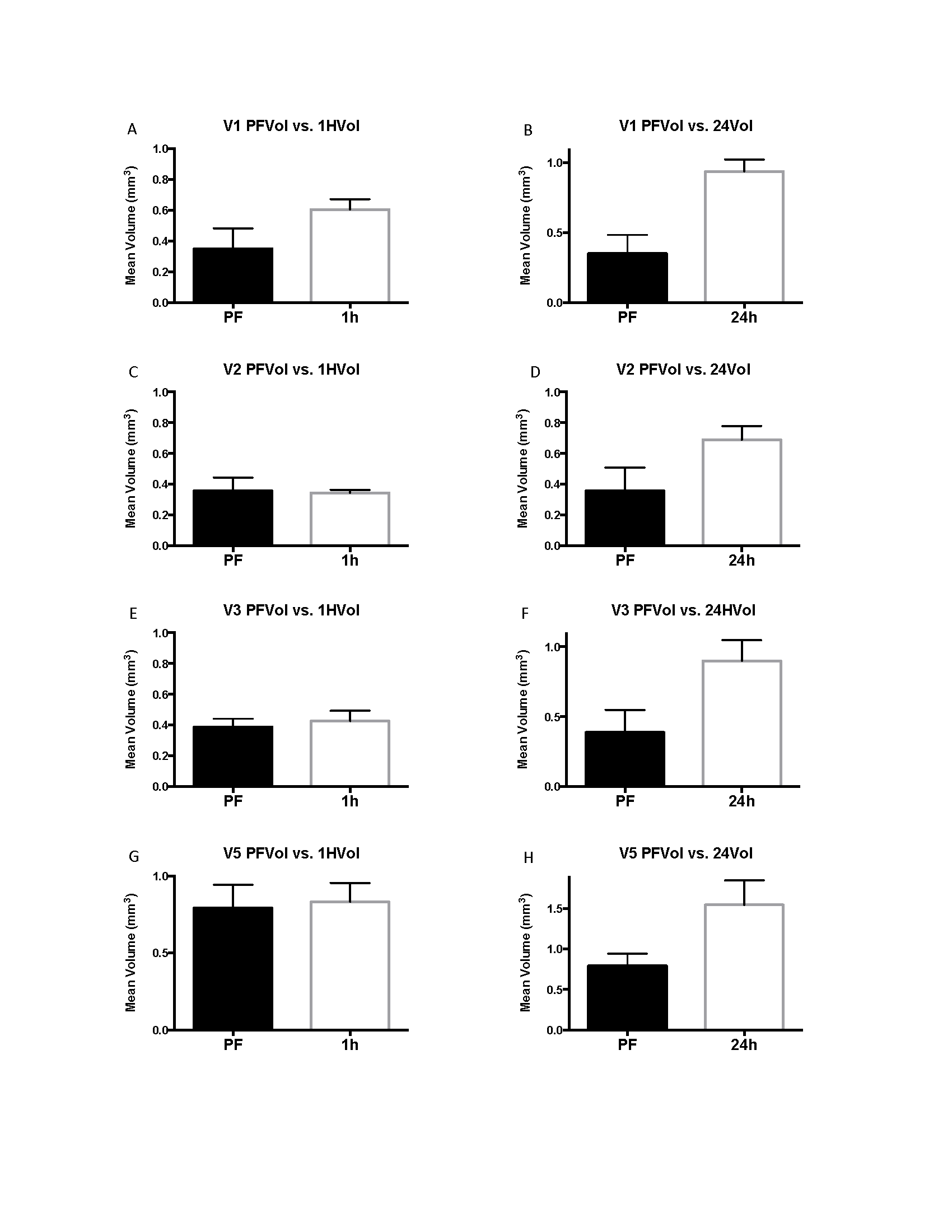

Supplement: S3 Fig — Mean volume (abbreviated Vol) is reported in mm3. The error bars represent standard error of the mean. PF refers to pre-flood cohorts, 1H refers to 1-hour cohorts, and 24h refers to 24-hour cohorts. (TIFF) [file pone.0223304.s003.tiff]

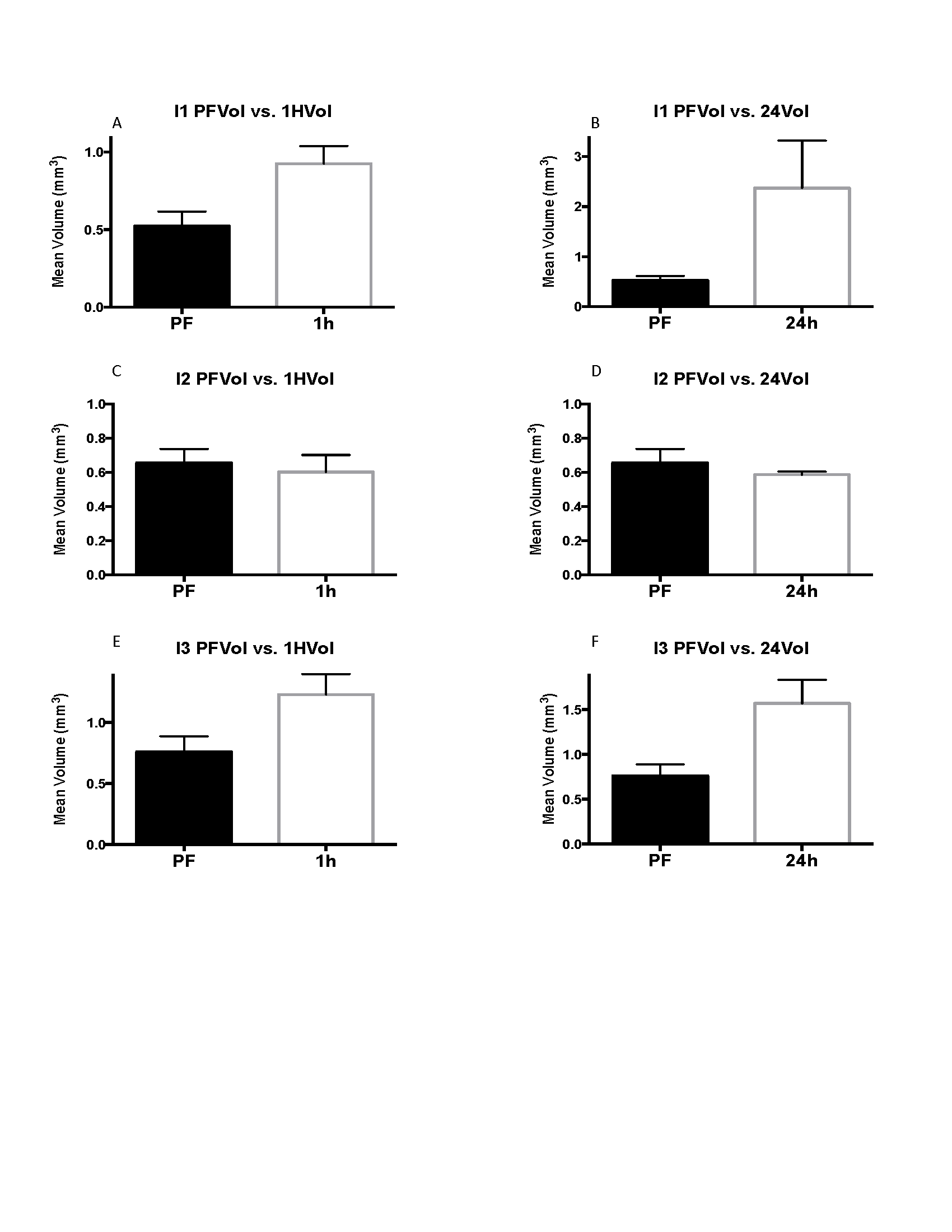

Supplement: S4 Fig — Mean volume (abbreviated Vol) is reported in mm3. The error bars represent standard error of the mean. PF refers to pre-flood cohorts, 1H refers to 1-hour cohorts, and 24h refers to 24-hour cohorts. (TIFF) [file pone.0223304.s004.tiff]
