# Supplementary material for: Effects of sea-level rise on physiological ecology of populations of a ground-dwelling ant
Source: PLoS One. 2020 Apr 17;15(4):e0223304. doi: 10.1371/journal.pone.0223304 (PMC7164625; doi:10.1371/journal.pone.0223304)
Supplement: S1 Table — Head width, stinger length, and head length are reported in mm. Volume is in mm3. N represents the number of workers within the corresponding group, P is the p-value, t is the t-statistic from unpaired t-tests, U is U-value from Mann-Whitney U tests, and df is the degrees of freedom. Tests that determined significant (p < 0.005) differences are marked by the word “yes” under the column labeled “different”. (PDF) [file pone.0223304.s005.pdf]

| | | | | | | | | | |
|---|---|---|---|---|---|---|---|---|---|
| I2 1-hour venom sac volume | Mann-Whitney | 0.60 ± 0.100 | 0.24-2.27 | 20 | 0.2447 | No | | 156.5 | |
| I2 24-hour venom sac volume | Mann-Whitney | 0.89 ± 0.090 | 0.41-1.80 | 20 | 0.8566 | No | | 193 | |
| | | | | | | | | | |
| I3 Pre-flood head width | | 0.84 ± 0.045 | 0.71-1.05 | 9 | | | | | |
| I3 1-hour head width | Mann-Whitney | 1.04 ± 0.049 | 0.78-1.32 | 10 | 0.0106 | Yes | | 14.5 | |
| I3 24-hour head width | Mann-Whitney | 1.19 ± 0.050 | 0.87-1.37 | 9 | 0.0001 | Yes | | 3 | |
| | | | | | | | | | |
| I3 Pre-flood venom sac volume | | 0.76 ± 0.127 | 0.30-1.47 | 9 | | | | | |
| I3 1-hour venom sac volume | Unpaired t-test | 1.23 ± 0.169 | 0.46-2.06 | 10 | 0.0435 | Yes | 2.181 | | 17 |
| I3 24-hour venom sac volume | Unpaired t-test | 1.57 ± 0.263 | 0.68-3.00 | 10 | 0.016 | Yes | 2.673 | | 17 |
