# Supplementary material for: Effects of sea-level rise on physiological ecology of populations of a ground-dwelling ant
Source: PLoS One. 2020 Apr 17;15(4):e0223304. doi: 10.1371/journal.pone.0223304 (PMC7164625; doi:10.1371/journal.pone.0223304)
Supplement: S2 Table — Head width, stinger length, and head length are reported in mm. Volume is in mm3. N represents the number of workers within the corresponding group, P is the p-value, and U is U-value from Mann-Whitney U tests. Tests that determined significant (p < 0.005) differences are marked by the word “yes” under the column labeled “different”. (PDF) [file pone.0223304.s006.pdf]

Large Coastal Pre-flood head width		1.12 ± 0.027	1.06-1.21	5			
Large Coastal 1-hour head width	Mann-Whitney	1.09 ± 0.024	1.02-1.22	7	0.4091	No	12
Large Coastal 24-hour head width	Mann-Whitney	1.21 ± 0.027	1.0-1.48	28	0.212	No	44.5
Large Coastal Pre-flood venom sac volume		1.05 ± 0.228	0.46-1.84	5			
Large Coastal 1-hour venom sac volume	Mann-Whitney	0.97 ± 0.148	0.31-1.53	7	0.8586	No	16
Large Coastal 24-hour venom sac volume	Mann-Whitney	1.58 ± 0.180	0.51-4.78	28	0.2215	No	45
