# Supplementary material for: Effects of sea-level rise on physiological ecology of populations of a ground-dwelling ant
Source: PLoS One. 2020 Apr 17;15(4):e0223304. doi: 10.1371/journal.pone.0223304 (PMC7164625; doi:10.1371/journal.pone.0223304)
Supplement: S3 Table — Vol/HW refers to mean relative value that results from dividing venom sac volume by head width and is reported in mm2. N represents the number of workers within the corresponding group, P is the p-value, and U is U-value from Mann-Whitney U tests. Tests that determined significant (p < 0.005) differences are marked by the word “yes” under the column labeled “different”. (PDF) [file pone.0223304.s007.pdf]

Large Coastal 1-hour Vol/HW	Mann-Whitney	0.89	0.139	0.29-1.40	7	0.8207	No	16
Large Coastal 24-hour Vol/HW	Mann-Whitney	1.26 ± 0.118		0.42-0.32	28	0.3198	No	50
