# Supplementary material for: Effects of sea-level rise on physiological ecology of populations of a ground-dwelling ant
Source: PLoS One. 2020 Apr 17;15(4):e0223304. doi: 10.1371/journal.pone.0223304 (PMC7164625; doi:10.1371/journal.pone.0223304)
Supplement: S4 Table — Equations are the form Y = mx +b where m is the slope and b is the y-intercept. R2 refers to the R–squared value, F refers to the F, and P is the p-value, which determines whether the slope is significantly non-zero. (PDF) [file pone.0223304.s008.pdf]

Factor(s) tested	Test	Slope ± SEM	Equation	R^2	F	P	Non-zero slope
Total Coastal head width	Linear regression	0.06 ± 0.020	Y = 0.06X + 0.73	0.05037	11.35	0.0009	Yes
Total Coastal volume	Linear regression	0.23 ± 0.055	Y = 0.23X + 0.38	0.09408	16.82	<0.0001	Yes
Total Coastal stinger length	Linear regression	0.03 ± 0.010	Y = 0.03X + 0.51	0.06032	10.4	0.0015	Yes
Total Coastal head length	Linear regression	0.08 ± 0.021	Y = 0.08X + 0.94	0.06862	15.77	<0.0001	Yes
Small Coastal head width	Linear regression	0.01 ± 0.01	Y = 0.01X + 0.64	0.05039	5.201	0.0247	Yes
Small Coastal volume	Linear regression	0.07 ± 0.02	Y = 0.07X + 0.33	0.07193	7.596	0.007	Yes
Medium Coastal head width	Linear regression	-0.01 ± 0.012	Y = -0.01X + 0.86	0.01491	1.12	0.2934	No
Medium Coastal volume	Linear regression	0.19 0.063	Y = 0.19X + 0.48	0.1145	9.572	0.0028	Yes
Large Coastal head width	Linear regression	0.06 ± 0.028	Y = 0.06X + 1.08	0.11	4.696	0.0366	Yes
Large Coastal volume	Linear regression	0.33 ± 0.19	Y = 0.33X + 0.88	0.07487	3.075	0.0875	No
