# Supplementary material for: Effects of sea-level rise on physiological ecology of populations of a ground-dwelling ant
Source: PLoS One. 2020 Apr 17;15(4):e0223304. doi: 10.1371/journal.pone.0223304 (PMC7164625; doi:10.1371/journal.pone.0223304)
Supplement: S5 Table — Head width, stinger length, and head length are reported in mm. Volume is in mm3. N represents the number of workers within the corresponding group, P is the p-value, and U is U-value from Mann-Whitney U tests. Tests that determined significant (p < 0.005) differences are marked by the word “yes” under the column labeled “different”. (PDF) [file pone.0223304.s009.pdf]

Total Inland 24-hour head length	Mann-Whitney	1.16 ± 0.038	0.76-1.75	50	0.0116	Yes	866
