# Supplementary material for: Effects of sea-level rise on physiological ecology of populations of a ground-dwelling ant
Source: PLoS One. 2020 Apr 17;15(4):e0223304. doi: 10.1371/journal.pone.0223304 (PMC7164625; doi:10.1371/journal.pone.0223304)
Supplement: S6 Table — Head width, stinger length, and head length are reported in mm. Volume is in mm3. N represents the number of workers within the corresponding group, P is the p-value, and U is U-value from Mann-Whitney U tests. Tests that determined significant (p < 0.005) differences are marked by the word “yes” under the column labeled “different”. (PDF) [file pone.0223304.s010.pdf]

Large Inland Pre-flood head width		1.12 ± 0.040	1.05-1.26	5			
Large Inland 1-hour head width	Mann-Whitney	1.14 ± 0.029	1.02-1.32	12	0.8153	No	27.5
Large Inland 24-hour head width	Mann-Whitney	1.20 ± 0.031	1.05-1.37	15	0.1741	No	21.5
Large Inland Pre-flood venom sac volume		1.47 ± 0.278	0.52-2.03	5			
Large Inland 1-hour venom sac volume	Mann-Whitney	1.59 ± 0.135	0.61-2.27	12	0.8196	No	27.5
Large Inland 24-hour venom sac volume	Mann-Whitney	1.73 ± 0.189	0.86-3.00	15	0.8155	No	34.5
