# Supplementary material for: Effects of sea-level rise on physiological ecology of populations of a ground-dwelling ant
Source: PLoS One. 2020 Apr 17;15(4):e0223304. doi: 10.1371/journal.pone.0223304 (PMC7164625; doi:10.1371/journal.pone.0223304)
Supplement: S7 Table — Head width, stinger length, and head length are reported in mm. Volume is in mm3. N represents the number of workers within the corresponding group, P is the p-value, and U is U-value from Mann-Whitney U tests. Tests that determined significant (p < 0.005) differences are marked by the word “yes” under the column labeled “different”. (PDF) [file pone.0223304.s011.pdf]

Factor(s) tested	Test	Mean ± SEM	Range	N	P	Different	t	U	df
Total Inland Pre-flood Vol/HW		0.76 ± 0.050	0.15-1.73	49					
Total Inland 1-hour Vol/HW	Unpaired t-test	0.93 ± 0.058	0.20-1.81	50	0.0252	Yes	2.273		97
Total Inland 24-hour Vol/HW	Mann-Whitney	1.03 ± 0.067	0.26-2.4	50	0.0019	Yes		785	
Small Inland Pre-flood Vol/HW		0.66 ± 0.055	0.19-1.16	25					
Small Inland 1-hour Vol/HW	Unpaired t-test	0.65 ± 0.062	0.20-1.20	18	0.9205	No	0.1104		41
Small Inland 24-hour Vol/HW	Mann-Whitney	0.65 ± 0.049	0.26-0.94	16	0.6965	No		185	
Medium Inland Pre-flood Vol/HW		0.74 ± 0.062	0.15-1.24	19					
Medium Inland 1-hour Vol/HW	Unpaired t-test	0.90 ± 0.063	0.36-1.53	20	0.0792	No	1.805		37
Medium Inland 24-hour Vol/HW	Unpaired t-test	1.04 ± 0.068	0.41-1.56	19	0.0028	Yes	3.204		36
Large Inland Pre-flood Vol/HW		1.29 ± 0.220	0.48-1.73	5					
Large Inland 1-hour Vol/HW	Mann-Whitney	1.40 ± 0.112	0.48-1.81	12	0.703	No		26	
Large Inland 24-hour Vol/HW	Mann-Whitney	1.44 ± 0.1417	0.78-2.4	15	0.8827	No		36	
