# Supplementary material for: Effects of sea-level rise on physiological ecology of populations of a ground-dwelling ant
Source: PLoS One. 2020 Apr 17;15(4):e0223304. doi: 10.1371/journal.pone.0223304 (PMC7164625; doi:10.1371/journal.pone.0223304)
Supplement: S8 Table — Vol/HW refers to mean relative value that results from dividing venom sac volume by head width and is reported in mm2. N represents the number of workers within the corresponding group, P is the p-value, and U is U-value from Mann-Whitney U tests. Tests that determined significant (p < 0.005) differences are marked by the word “yes” under the column labeled “different”. (PDF) [file pone.0223304.s012.pdf]

Factor(s) tested	Test	Slope ± SEM	Equation	R^2	F	P	Nonzero
Total Inland head width	Linear regression	0.06 ± 0.018	Y = 0.06X + 0.79	0.06676	10.52	0.0015	Yes
Total Inland volume	Linear regression	0.19 ± 0.055	Y = 0.19X + 0.64	0.07772	12.39	0.0006	Yes
Total Inland stinger length	Linear regression	0.01 ± 0.001	Y = 0.01X + 0.56	0.01793	2.684	0.1035	No
Total Inland head length	Linear regression	0.07 ± 0.022	Y = 0.07X + 1.03	0.06287	9.862	0.002	Yes
Small Inland head width	Linear regression	0.00 ± 0.00	Y = 0.00X + 0.70	0.0114	0.6575	0.4208	No
Small Inland volume	Linear regression	0.01 ± 0.028	Y = -0.01X + 0.46	0.0008211	0.179	0.8294	No
Medium Inland head width	Linear regression	0.02 ± 0.012	Y = 0.02X + 0.82	0.06087	3.63	0.0619	No
Medium Inland volume	Linear regression	0.15 ± 0.044	Y = 0.15X + 0.61	0.1754	11.91	0.0011	Yes
Large Inland head width	Linear regression	0.04 ± 0.026	Y = 0.04X + 1.11	0.07111	2.297	0.1401	No
Large Inland volume	Linear regression	0.13 ± 0.150	Y = 0.13X + 0.46	0.02563	0.789	0.3815	No
